# Internalization of a polysialic acid-binding *Escherichia coli* bacteriophage into eukaryotic neuroblastoma cells

**DOI:** 10.1038/s41467-017-02057-3

**Published:** 2017-12-04

**Authors:** Timo A. Lehti, Maria I. Pajunen, Maria S. Skog, Jukka Finne

**Affiliations:** 1 Division of Biochemistry and Biotechnology, Department of Biosciences, University of Helsinki, P.O. Box 56, FI-00014 Helsinki, Finland; 2 Present Address: Department of Bacteriology and Immunology, Medicum, Research Programs Unit, Immunobiology, University of Helsinki, P.O. Box 21, FI-00014 Helsinki, Finland

## Abstract

Eukaryotic organisms are continuously exposed to bacteriophages, which are efficient gene transfer agents in bacteria. However, bacteriophages are considered not to pass the eukaryotic cell membrane and enter nonphagocytic cells. Here we report the binding and penetration of *Escherichia coli* PK1A2 bacteriophage into live eukaryotic neuroblastoma cells in vitro. The phage interacts with cell surface polysialic acid, which shares structural similarity with the bacterial phage receptor. Using fluorescence and electron microscopy, we show that phages are internalized via the endolysosomal route and persist inside the human cells up to one day without affecting cell viability. Phage capsid integrity is lost in lysosomes, and the phage DNA is eventually degraded. We did not detect the entry of phage DNA into the nucleus; however, we speculate that this might occur as a rare event, and propose that this potential mechanism could explain prokaryote–eukaryote gene flow.

## Abstract (web summary)

Eukaryotic organisms are continuously exposed to bacteriophages, but these are not thought to enter non-phagocytic cells. Here, Lehti et al. show that a bacteriophage can bind to a specific receptor on the surface of human neuroblastoma cells in vitro, and be internalized via the endolysosomal route.

## Introduction

The evolution of cellular life is tightly bound to viruses that use host organisms to complete their life cycle. Bacteriophages, viruses that infect bacteria, are the most numerous replicating entities in the biosphere, with an estimated global population of 10^31^ phage particles^1, 2^. Phages play fundamental roles in bacterial ecology and virulence^3^. Their ability to package DNA fragments of the host genome during phage propagation makes them powerful vehicles for horizontal gene transfer, a dominant process in microbial evolution^4^. It has been estimated that phages mediate over 10^16^ gene transfer events each second^5^. In the face of omnipresent phage-rich environments, animals frequently come into contact with phages. Host mucosal surfaces are densely populated by residential microbial communities that consist largely of bacteria. Within this setting, the phage populations are dominating the viral community in the gut^6, 7^ and have an important contribution to bacterial–host interactions^8, 9^.

Single observations suggest that interdomain genetic exchanges from bacteria to eukaryotes have occurred during evolution^10–12^. Bacterium-to-eukaryote horizontal gene transfer events are suggested to provide novel traits important in conferring advantages for specific niches, such as genes encoding metabolic enzymes^13, 14^. However, the mechanisms that permit the acquisition of genetic variability via interdomain transfers remain elusive. The cell membrane acts as a barrier between the aqueous cytoplasm and the outside environment, and this efficiently delimits the transfer of molecules, such as DNA, across the membrane. Unlike prokaryotes, eukaryotes lack mechanisms for uptake of free DNA from the environment. While it is generally assumed that the enormous reservoir of genetic diversity encompassed by phages is restricted within the borders of the prokaryotic world, evidence is accumulating that gene flow through phages is potentially a horizontal gene transfer pathway between prokaryotes and eukaryotes^15–17^. In line with this, phage genes have under experimental conditions been integrated into the genome of eukaryotic cells^18^. Phage genes can also be expressed in eukaryotic cells^19–21^.

While it has been previously shown that phage lambda is capable of transducing mammalian cells^20, 21^, there is currently no direct evidence demonstrating a specific mechanism by which phages traverse the eukaryotic membrane and enter nonphagocytic cells, and thereby open a door for gene transfer. Here, we show that bacteriophage bound specifically to a mammalian cell receptor can pass the cell membrane barrier and be internalized by means of endocytic vesicles. The access to the cell could conceivably provide an entry port for the introduction of foreign genetic material into the cell, even though we did not detect the entry of phage DNA into the cell nucleus. The phage–eukaryotic cell interaction reported here expands the functional capacity of phages and support that phages represent an unexplored factor in the evolution of eukaryotes.

## Results

### Binding of bacteriophage to a target on neuroblastoma cells

The *Escherichia coli* bacteriophage PK1A2, a member of the *Podoviridae* family and variant of PK1A, was originally isolated by its ability to bind bacteria containing reduced amounts of its polysaccharide receptor, the K1 polysialic acid capsule^22^ consisting of α2,8-linked N-acetylneuraminic acid units. The bacterial receptor structure is identical to polysialic acid present on mammalian cells^23^ and protects the bacterial cell against the immune system during invasive infection^24^. Compared to the PK1A phage with catalytic endosialidase as a tailspike protein, PK1A2 has two amino acid substitutions in the endosialidase that abolish the catalytic activity but still permit polysialic acid binding^25^. PK1A2 phage is able to recognize and remain bound to polysialic acid on paraformaldehyde-fixed baby hamster kidney fibroblast cells and tissue sections of developing rat brain^26^. In eukaryotes, polysialic acid is highly expressed in the nervous system during development, but also detected in malignancies such as neuroblastomas^27, 28^. As receptor–ligand interaction is the initial step of viral infection, we first examined the interaction of PK1A2 with human cell lines expressing different amounts of polysialic acid to confirm the specificity of the phage binding.

**Fig. 1:**
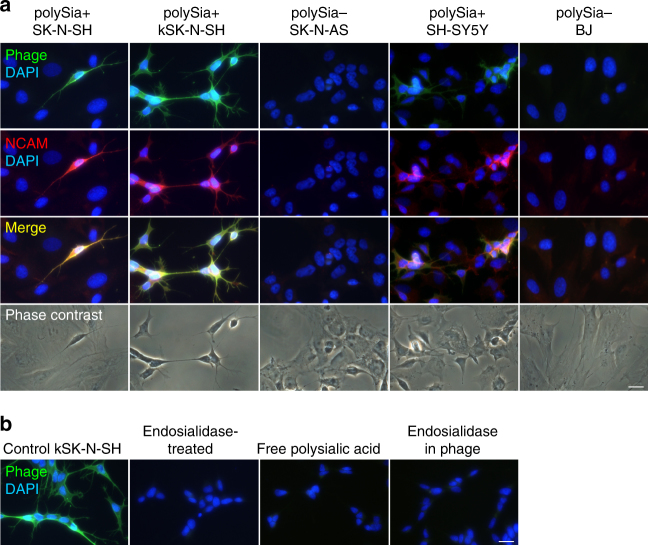
Bacteriophage PK1A2 binds specifically to mammalian polysialic acid-expressing cells. **a** Fluorescence microscopic images of cells incubated with FITC-labeled PK1A2 phages (green) and neural cell adhesion molecule NCAM antibodies (red) to assess phage surface binding. Polysialic acid-containing (polySia+) human neuroblastoma SK-N-SH, kSK-N-SH and SH-SY5Y cells as well as human polysialic acid-negative (polySia−) neuroblastoma SK-N-AS and fibroblast BJ cells were incubated with FITC-labeled phages for 1 h at room temperature and stained for NCAM, the carrier protein of polysialic acid. **b** Inhibition of phage binding to kSK-N-SH cells by pretreatment with endosialidase or incubation in the presence of free polysialic acid. As a control, phage containing catalytically active endosialidase as the binding agent was used. Nuclei were stained with DAPI (blue). Representative images from two to three biological replicates are shown. The scale bars represent 20 µm

We used epifluorescence microscopy to evaluate the binding of PK1A2 to cultured human cells. In order to examine cell binding, we labeled purified phage particles with fluorescein isothiocyanate (FITC) and added them to SK-N-SH cells, a human neuroblastoma cell line that expresses polysialic acid as a part of the neural cell adhesion molecule NCAM^29^. This continuous cell line is commonly used as an in vitro model in neuroscience and polysialic acid research. In culture, the cell line contains two morphological variants, the neuroblastic (N-type) cells rich in polysialic acid as well as the flat substrate-adherent/Schwannian (S-type) cells containing little polysialic acid^30^. A polysialic acid and NCAM-expressing N-type cell population (kSK-N-SH) has been previously isolated from the SK-N-SH cell line^31^. The labeled PK1A2 phages bound specifically to the N-type cells and had a similar distribution to the polysialic acid carrier molecule NCAM (Fig. 1a). Similar results were obtained with SH-SY5Y cells that also express polysialic acid^32^, whereas the S-type cells of SK-N-AS and human BJ fibroblasts, which contain little or no polysialic acid, were negative for phage binding, and had low or undetectable amounts of NCAM (Fig. 1a).

To verify that the phages bind specifically to polysialic acid, we assessed phage binding after removal of cell surface polysialic acid by endosialidase, an enzyme that specifically degrades polysialic acid^33^. The binding of phages to the endosialidase-treated cells was abolished (Fig. 1b). The binding was also inhibited by competition with a high concentration of soluble polysialic acid (purified polysialic acid from *E. coli* K1). In parallel control experiments, FITC-labeled PK1A phages which differ from the PK1A2 phage in expressing active endosialidase themselves did not remain bound to the cells (Fig. 1b) because of degrading their polysialic acid-containing target^26^.

### Internalization of bacteriophage into cells

**Fig. 2:**
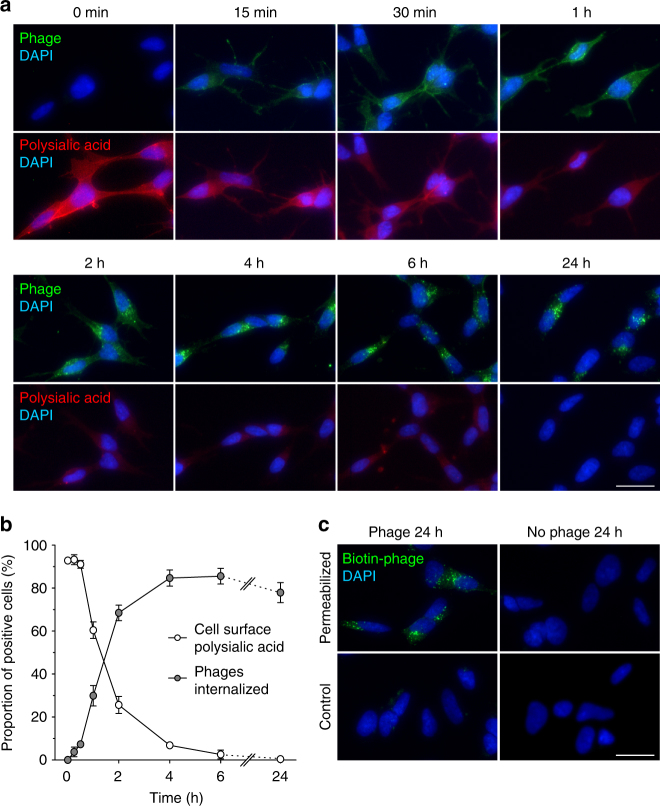
Phages are endocytosed into live neuroblastoma cells. **a** Time course of PK1A2 phage internalization into cultured kSK-N-SH cells. Cells were incubated with FITC-labeled phages (green) at 37 °C for the times indicated, fixed and examined for the presence of phages and surface-expressed polysialic acid (red). **b** Quantitative analysis of phage internalization. Data are presented as the percentages of positive cells that stained for cell surface polysialic acid or contained internalized phages and the values represent means of three randomly chosen fields ± s.d. of a single experiment. For each field, at least 60 cells were examined. **c** Immunofluorescence detection of internalized biotin-conjugated phages after 24 h of incubation. Control cells or cells permeabilized with 0.2% Triton X-100 were stained with Alexa Fluor 488 FluoroNanogold-streptavidin (green). Cells without phages were used as control of staining specificity. Nuclei were stained with DAPI (blue). All data shown are representative for two biological replicates. The scale bars represent 20 µm

We next examined whether the phage binds to live cells and is internalized into the cells. Live kSK-N-SH cells were incubated with the phage particles at 37 °C for various intervals after which the cells were fixed and examined. After 30 min of incubation, clusters of fluorescent phages started to appear in the cell and their amount increased up to 24 h (Fig. 2a, b). The appearance of phages inside the cell correlated with a decline in the amount of available polysialic acid ligand at the cell surface (Fig. 2a, b), which remained at the cell surface if no phage was added (Supplementary Fig. 1). After 6 h, cell surface polysialic acid had disappeared and the phages had redistributed to vesicle-like structures in the perinuclear region of the cells. The results suggest that the binding induces internalization of the phage–polysialic acid complex and consequently leads to the progressive disappearance of polysialic acid from the cell surface.

To confirm that the phages were indeed localized inside the cell, cells incubated with biotin-labeled phages were probed with a streptavidin-conjugated stain. We found that the phages could only be observed in cells made permeable to the stain (Fig. 2c), which suggests that the phages resided intracellularly and were not accessible in non-permeabilized cells. Altogether, the results indicate that binding of the phages to polysialic acid is followed by their progressive internalization into the cells.

### Polysialic acid dependence of bacteriophage internalization

**Fig. 3:**
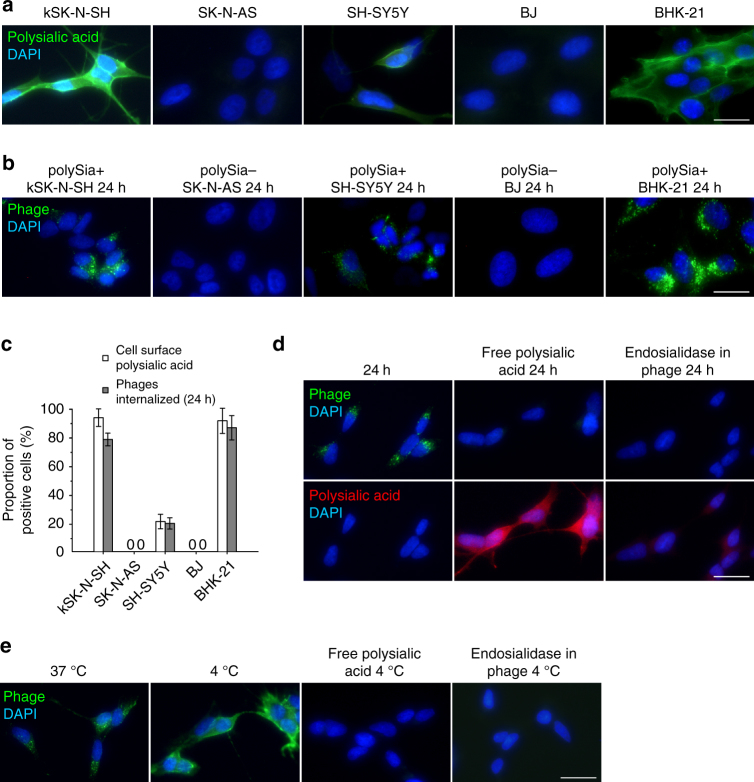
Phage internalization requires polysialic acid. **a** Detection of cell surface polysialic acid in human neuroblastoma (kSK-N-SH, SK-N-AS and SH-SY5Y), human fibroblast (BJ) and hamster kidney (BHK-21) cells. The cells were grown on coverslips, fixed and stained for surface-expressed polysialic acid. **b** Internalization of FITC-labeled PK1A2 phages into polysialic acid-containing (polySia+ ) cells compared to polysialic acid-negative (polySia−) cells after 24 h of incubation at 37 °C. **c** Quantitative analysis of phage internalization. Percentages of cells expressing cell surface polysialic acid or having endocytosed FITC-labeled phages after incubation with phages for 24 h at 37 °C. The values represent means of three randomly chosen fields ± s.d. of a single experiment. For each field, at least 25 cells were examined. **d** Inhibition of phage internalization. kSK-N-SH cells incubated with phages (green) in the absence or the presence of free polysialic acid, or control phages containing active endosialidase for 24 h at 37 °C. After incubation, the cells were fixed and stained for surface-expressed polysialic acid (red). **e** Effect of low temperature on phage internalization. kSK-N-SH cells were incubated with phages in the absence or the presence of free polysialic acid for 4 h at 37 or 4 °C, or control phages containing active endosialidase for 4 h at 4 °C. Nuclei were stained with DAPI (blue). All data shown are representative for two biological replicates. The scale bars represent 20 µm

Intracellular phages were present only in polysialic acid-expressing cells and not in polysialic acid-negative cells (Fig. 3a–c). Incubation of kSK-N-SH cells together with free polysialic acid prevented phage internalization and retained polysialic acid at the cell surface (Fig. 3d). Control PK1A phages containing catalytically active endosialidase caused the removal of polysialic acid from the cell surface and were not internalized.

We also examined whether phage internalization could be arrested by low temperature, which is known to block endocytosis. Incubating the cells at 4 °C allowed phage binding to the cells but inhibited phage internalization (Fig. 3e). As controls, soluble bacterial polysialic acid abolished the binding of the phage, and endosialidase-containing phage (due to its polysialic acid-cleaving activity) did not remain bound (Fig. 3e).

### Endolysosomal routing of bacteriophage

To characterize the intracellular trafficking of phages in kSK-N-SH cells, it was necessary to distinguish between surface-bound and internalized phage particles. Proteolytic treatments used to remove or degrade extracellular phages, subtilisin^34^, trypsin^35^ and proteinase K^36^, were not effective or only partially in the logarithmic scale of phage titering (up to a 3-log decrease of phage titer for proteinase K at 20 mg ml^−1^). Even the removal of non-internalized, surface-bound phages by acid wash^37^ was not efficient enough and also affected cell morphology. In contrast, when free polysialic acid was used to displace the bound phages from the cell surface by competition, complete removal of phages was observed, with no apparent morphological effects on the cells (Supplementary Fig. 2). Vesicles containing phages were still present visible in the perinuclear location.

**Fig. 4:**
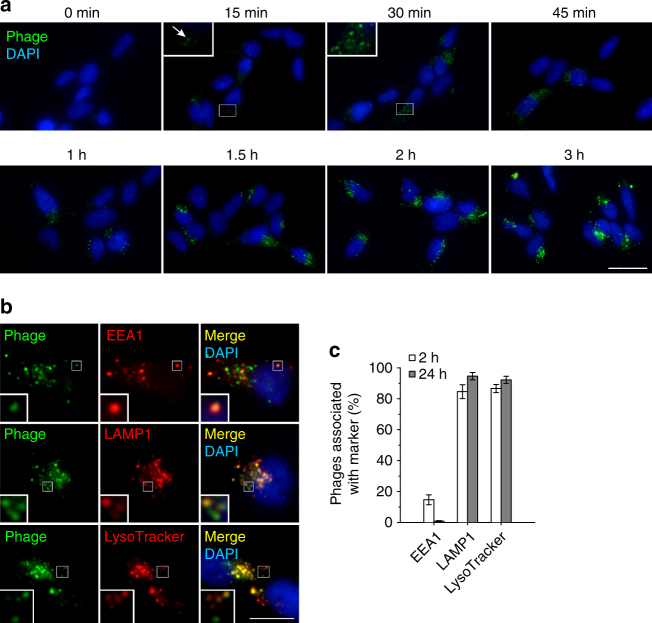
Progression of phage internalization after removal of surface-bound phages and direction to lysosomes. **a** Time course of PK1A2 phage internalization into kSK-N-SH cells. Following incubation with FITC-labeled phages (green) for the times indicated at 37 °C, the non-internalized phages were stripped off by polysialic acid competition. An intracellular phage cluster is indicated with an arrow. **b** Phage clusters in early-endosomal and lysosomal compartments. kSK-N-SH cells were incubated with FITC-labeled phages (green) for 2 h at 37 °C, extracellular phages were removed by polysialic acid competition, and the fixed cells were immunostained for the early-endosomal marker EEA1 or late endosomal/lysosomal marker LAMP1 (red). The lysosomal stain LysoTracker (red) was added to the medium 30 min before the end of incubation with phage. **c** Quantification of phage clusters positive for EEA1, LAMP1 or LysoTracker. Following incubation with FITC-labeled phages for 2 or 24 h at 37 °C, the cells were subjected to immunostaining as in **b**. The data represent the mean ± s.d. of two independent experiments depicting the results from at least 300 vesicles for each condition. In all experiments nuclei were stained with DAPI (blue). Representative images from two to four biological replicates are shown. The scale bars represent 20 µm in **a**, 10 µm in **b**, the insets have been enlarged 3-fold

After removal of surface-bound phages, a low level of fluorescence could be detected in intracellular vesicular compartments already after 15 min incubation (Fig. 4a). As also seen in Fig. 2, increasing the incubation time enhanced the intracellular accumulation and clustering of phage particles, which suggested that the formation of the intracellular clusters of phages is a result of constitutive endocytosis.

To determine whether internalized phage particles are shuttled to the endolysosomal pathway, phage-incubated cells were stained for early endosomes (early endosome antigen 1, EEA1) and late endosomes/lysosomes (lysosome associated membrane protein 1, LAMP1). After 2 h, a minor portion of phages was associated with early endosomes, whereas most phages were found in the late endosomal/lysosomal compartments (Fig. 4b, c). In line with this, the majority of phages was associated with lysosomes labeled with the acidotropic dye LysoTracker (Fig. 4b, c).

### Persistence and delayed inactivation of internalized phage

**Fig. 5:**
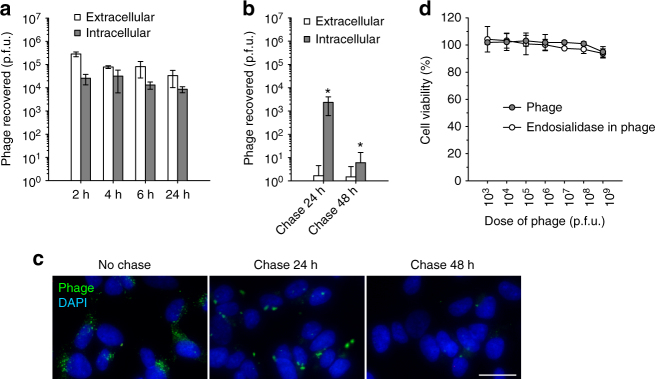
Persistence and inactivation of internalized phage. **a** Recovery of internalized or cell surface bound PK1A2 phages from kSK-N-SH cells. Cells were incubated with phages at 37 °C for the times indicated. The amounts of extracellular and intracellular phages were determined and are shown as plaque-forming units (p.f.u.). Each bar represents the mean ± s.d. of three independent experiments. **b** Inactivation of internalized phages in the cells. Cells were pulsed with phages for 24 h at 37 °C, after which extracellular phages were removed by polysialic acid competition (last column in **a**). Subsequently, the phages were chased with cell medium without phages for the times indicated. Phage quantitation results are expressed as mean ± s.d. of three independent experiments. The asterisks mark *P*-value of < 0.03 (compared to last column in **a**) as calculated by Student’s *t*-test. **c** Visualization of phages in pulse-chase experiment. Cells were pulsed with FITC-labeled phages (green) and chased with phage-free cell medium as in **a** and **b**, respectively. Nuclei were stained with DAPI (blue). Representative images from two biological replicates are shown. The scale bar represents 20 µm. **d** Absence of cellular cytotoxicity of phage. kSK-N-SH cells were incubated with increasing doses of phage or endosialidase-containing control phage in the growth medium for 24 h and subjected to cell viability assay. The data represent the mean ± s.d. of three independent experiments including two technical replicates each

To determine if phages that have been internalized by the cells remained infectious, we performed a quantitative analysis of intracellular and extracellular phage populations at different time points. In order to recover only internalized phage particles from kSK-N-SH cells, the phages bound to the surface were removed by polysialic acid competition, after which the intracellular phages were released by lysing the cells. The samples were then used to infect their host bacteria for quantification of the recovered phages by titration analysis. To quantify cell surface-bound phages, the polysialic acid treatment was omitted and non-lysed detached cells were used. The results revealed that a notable proportion of the internalized phages retained their infectious capacity during the 24 h incubation period (Fig. 5a). At 24-h time point, ~30% of the intracellular phages were still infectious as compared to the titer at 2 h.

To further study the stability of the phage inside the kSK-N-SH cell, we performed a pulse-chase experiment. After incubating the phages with cells for 24 h, the extracellular and surface-bound phages were removed by polysialic acid competition, and the internalized phages were chased for additional 24 and 48 h in culture media without phages. A quarter of the intracellular phages remained active during the first 24-h chase (Fig. 5b). On the contrary, a dramatic 3-log reduction was observed after the next 24 h, resulting in a virtual disappearance of infectious phages from the cells. Control quantification of the number of any remaining extracellular phages showed that the polysialic acid treatment had efficiently removed any background noise due to non-internalized phages. Fluorescence microscopy confirmed that phages could be detected in the form of large clusters after 24-h chase, whereas almost no fluorescence signal was present at the end of the 48-h chase (Fig. 5c). Finally, cell viability assays were performed to assess whether the internalized phages could be toxic to the kSK-N-SH cells. The cells were exposed to increasing amounts of phages in the growth medium and the proportions of surviving cells after 24 h of incubation were determined. No apparent cytotoxicity to the cells was observed (Fig. 5d). Similar results were also obtained with the endosialidase-containing control phage which depolymerizes polysialic acid and remains extracellular.

### Phage DNA internalization and exposure

**Fig. 6:**
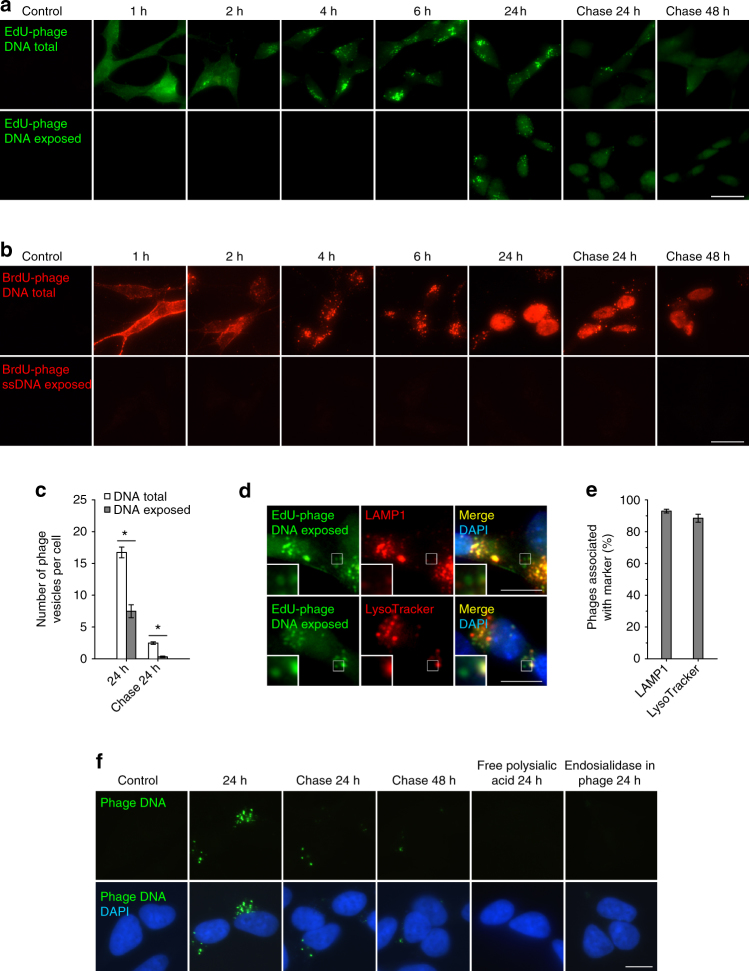
Time course of the exposure of phage DNA inside the cell. **a**, **b** Internalization of DNA-labeled PK1A2 phages into cultured kSK-N-SH cells at 37 °C. EdU (**a**) or BrdU (**b**) labeled phages (green and red, respectively) were detected under the conditions showing total DNA or exposed DNA as described in Methods. EdU staining reveals DNA irrespective of strand form, the BrdU method reveals DNA in single-strand form. Representative images from three biological replicates are shown. **c** Quantification of phage vesicles positive for EdU at 24 h and after subsequent chase for 24 h (each vesicle may contain several phage particles). At least 100 cells were quantified for each condition. Results are expressed as mean ± s.d. of three independent experiments. The asterisks mark *P*-value of < 0.005 as calculated by Student’s *t*-test. **d** Association of EdU-labeled phage clusters with lysosomal compartments. The cells were incubated with phages (green) for 24 h at 37 °C to reveal exposed phage DNA and LAMP1 (red) or LysoTracker (red) as in Figs. 4b and 6a. Nuclei were stained with DAPI (blue). Representative images from two biological replicates are shown. **e** Quantification of EdU-labeled phage vesicles positive for LAMP1 or LysoTracker at 24 h in **d**. At least 300 vesicles were analyzed for each condition and the data represent the mean ± s.d. of two independent experiments. **f** Detection of internalized phage DNA by fluorescence in situ hybridization (FISH). kSK-N-SH cells were incubated with phages in the absence or the presence of free polysialic acid or with control phages containing active endosialidase at 37 °C for the times indicated. The samples were processed for in situ hybridization using Alexa Fluor 488-labeled DNA probes covering the phage genome (green). Nuclei were stained with DAPI (blue). Representative images from three biological replicates are shown. The scale bars represent 20 µm in **a** and **b**, 10 µm in **d** and **f**, the insets have been enlarged 3-fold

To monitor the fate of the double-stranded phage DNA in the cell, we labeled it by incorporation of 5-ethynyl-2′-deoxyuridine (EdU) in the phage-*E. coli* culture. Using purified EdU-labeled phages, a progressive internalization of the phages from the cell surface was observed (Fig. 6a, upper panel). Chasing for additional 24 and 48 h led to a decrease and disappearance of the label, comparable to the result seen for the FITC-labeled phages (Fig. 5c). Similar results were obtained with phages labeled with 5-bromo-2′-deoxyuridine (BrdU) (Fig. 6b, upper panel).

The EdU-labeled phage particles provided a strong signal in this staining protocol only after acid pretreatment which opens the protein capsid and renders the phage genome accessible to the labeling reaction (Supplementary Fig. 3a). To detect exposed phage DNA without intact capsid, the staining was also performed without the acid pretreatment. In this case, no staining was detected up to 6 h of internalization, which indicated that the capsid was intact (Fig. 6a, lower panel). A punctate staining was observed in the perinuclear region at 24 h and gradually disappeared during the chase period.

In addition to the opening the capsid, the BrdU detection method requires that the double-stranded phage DNA has been denatured to the single-stranded form^38^ (Supplementary Fig. 3b). Cells incubated with BrdU-labeled phages not pretreated with acid gave no signal at any time point (Fig. 6b, lower panel), which indicated that phage DNA was not single-stranded, not even at the time point of exposure (24 h and onwards).

### Fate of phage DNA

At 24 h, as much as 45% of the intracellular EdU-labeled phages were detected without acid pretreatment, which suggests that the phage DNA was exposed (Fig. 6c). In order to find out the localization of these phages, their distribution in relation to the endocytic markers was investigated. The majority of the phage DNA was co-distributed with the late endosomal/lysosomal compartments (Figs. 6d, e). However, provided that the markers had reached all of the vesicles, a minor fraction of the particles with exposed DNA seemed not to be associated with these compartments (Fig. 6d, insets).

We next investigated the localization of internalized phage DNA sequences by fluorescence in situ hybridization (FISH) using a whole-genome approach. At 24 h and subsequent chase time points, punctate signals of phage DNA were found mostly in a perinuclear localization (Fig. 6f), in agreement with the results for total phage DNA staining using EdU and BrdU. We did not detect phage DNA in the cell nucleus using FISH.

Notably, EdU and BrdU signals often co-localized with DAPI staining in most cells at 24 h and onwards, which indicated that the nucleotides released from the internalized phages had reached the nuclei (Fig. 6a, b; Supplementary Fig. 4a, b). Similar staining was obtained if cells were cultured in the presence of free BrdU (Supplementary Fig. 5). In general, free nucleoside analogs are known to be incorporated into newly synthesized DNA during the growth of cells^39^.

### Detection of internalized phage by electron microscopy

**Fig. 7:**
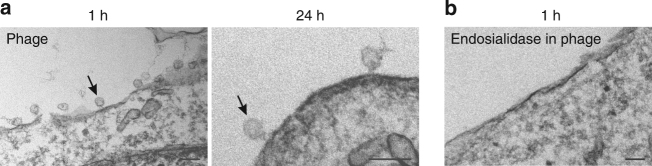
Ultrastructural analysis of cells with phages. **a**, **b** Transmission electron micrographs of thin sections of kSK-N-SH cells incubated with phages (**a**) or endosialidase-containing control phages (**b**) at 37 °C for the times indicated. The arrows indicate examples of surface-bound phage particles. The scale bars represent 100 nm

We further used transmission electron microscopy to examine the binding and internalization of the phage into the cells. At 1 h post-incubation, phage particles with their typical icosahedral-shaped capsid and short tail morphology were readily observed at the cell surface (Fig. 7a), whereas endosialidase-containing control phages were not observed interacting with the cells (Fig. 7b). As seen in Fig. 7a, surface-bound phage particles were still visible after 24 h incubation, albeit to a lesser extent than at 1 h. Structures similar in size and morphology to phage particles were found embedded in vesicles, however, it was not possible to identify them with certainty as phages due to their insufficient contrast.

**Fig. 8:**
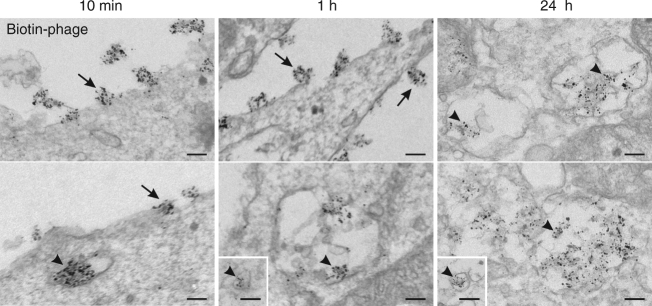
Ultrastructural visualization of internalized phages. Transmission electron micrographs of thin sections of kSK-N-SH cells incubated with biotin-conjugated PK1A2 phages at 37 °C for the times indicated. After incubation, the cells were fixed and stained with Alexa Fluor 488 FluoroNanogold-streptavidin probe, followed by silver-enhanced nanogold and gold toning treatments. Phage particles decorated with enhanced gold particles are seen at the cell surface (arrows) and within cytoplasmic vesicles (arrowheads). The scale bars represent 100 nm

To visualize the internalized phages, the phage was first labeled with biotin, incubated with the cells and then detected with streptavidin conjugated to Alexa Fluor 488 dye and Nanogold particles. Electron microscopy demonstrated surface-bound particles along the plasma membranes as well as intact phage particles enclosed in intracellular vesicles (Fig. 8). Most phage particles were found within vesicles containing several phages, some occasional vesicles a single phage particle were also observed. Consistent with the fluorescence microscopy results, the phages appeared to accumulate into large vesicles over time, as a result of vesicle fusion during their routing via the endolysosomal pathway.

## Discussion

The barrier function of the cell membrane keeps prokaryotic and eukaryotic gene pools separated. Despite this, a number of horizontal gene transfer events from bacteria to eukaryotes are indicated to have occurred in diverse eukaryotic groups and include both endosymbionts and nonendosymbionts^11, 13, 40^. The mechanisms involved are not known. Here, we demonstrate that phages can specifically interact with a cell surface component of eukaryotic cells, and that the bound phages can enter the cells.

As being by far the most abundant viruses in the human body, bacteriophages are integral components of our microbiota and serve as extensive reservoirs of genetic diversity^41^. Aside from the paradigm that phages can only infect bacteria and have no intrinsic tropism for other organisms, there are few indications that natural phage particles have direct cellular interactions with mammalian cells^26, 42^. Phage particles are able to pass through the intestinal mucosa to the bloodstream, spread systemically and translocate to tissues and organs such as the spleen^42–45^. The underlying mechanisms are not known but have been suggested to include breaks in the epithelial barriers or transepithelial transport by dendritic cells. Phages can even accumulate in the brain, at least when given intraperitoneally or intranasally^45–47^. Although it has been discussed that mammals may have mechanisms for uptake of phage particles^8, 18, 48, 49^ and interactions are indirectly indicated by lambda phage transduction^20, 21^, no detailed mechanism has been presented demonstrating that natural phages can enter nonphagocytic mammalian cells. With a polysialic acid-binding phage and mammalian cell lines, we observe in this study that phages are capable of penetration into the cells after specific binding to the cell surface. This demonstrates a gate to cross the boundaries between the bacterial virosphere and eukaryotes.

Similar to most eukaryotic viruses^50^, the PK1A2 phage seems to take advantage of the endocytic machinery of the eukaryotic cell for virus entry. It has been shown that the turnover of cell surface polysialic acid involves internalization of the polysaccharide and its protein carrier NCAM into endosomes^51^. Phage internalization reduces the amount of cell surface polysialic acid and this may have significant biological influences due to the role of this surface component as a regulator of cell interactions^28^. The entry process of the phage is temperature-dependent and is initiated by recognition and binding to the cellular polysialic acid, triggering endocytosis. The internalization results in the localization of the majority of the phages in lysosomes in the perinuclear region. In the lysosomes the phage capsid integrity is lost and the phage DNA is detected in its exposed form. The entry of phage DNA into the nucleus would in an evolutionary perspective be the next decisive step but is probably a rare event, which we were not able to detect under the experimental conditions used. Most phage DNA in the lysosomes is degraded and the nucleosides are reused by the cell, as indicated by the diffuse staining pattern of the marker nucleosides in the nuclei. It should be noted that foreign intracellular double-stranded DNA may have biological effects, such as modulation of the immune system^8, 48, 49^. In the case of Toll-like receptors, even DNA internalized into the lysosomal compartment induces signaling cascades and lysosomes in general are engaged in important regulatory functions^52^.

The structural similarity of polysialic acid polysaccharide of the bacterial host *E. coli* K1 and the polysialic acid at the surface of the eukaryotic cell lines of this study explains the ability of the phage to bind to both cell types. Thus the phage binding and internalization is based on the molecular mimicry between the pathogenic bacterium and its host. The PK1A2 phage represents a natural variant of the PK1A phage, which was obtained without mutagenesis by selection for binding to bacteria with reduced amount of polysialic acid^22^. Phage uptake was observed in this study with one specific phage and three mammalian cell lines in vitro. However, there are also other examples of structurally similar surface carbohydrates of pathogenic bacteria shared by the host cells such as the hyaluronan capsule of group A *Streptococcus* ^53^, sialylated capsular polysaccharide of group B *Streptococcus* ^54^, as well as sialylated lipooligosaccharide of *Campylobacter jejuni* ^55^ and meningococci^56^. Considering the prevalence of molecular mimicry in the microbial world^57, 58^, it is likely that there are many other host-mimicking cell surface epitopes that are targeted by phages. In addition, considering the enormous amount of phages present in the biosphere (~10^31^) and their natural susceptibility to genetic variation, it should be considered that a number of binding sites compatible with eukaryotic cell surface epitopes may be naturally generated by chance. Thus, there could be many more incidences of the uptake of phages by eukaryotic cells.

The phages’ ability to cross the physical barrier of eukaryotic cell represents a potential route for genetic exchanges, expanding the genetic pool accessible to eukaryotes. Recently, bacterial aerolysin and lysozyme genes were found to have been transferred multiple times into various eukaryotes^59, 60^. In both examples homologs were identified in phages, which suggests that they may have contributed to the distribution of the genes between bacteria and eukaryotes. Homologs of a capsid protein gene from *Chlamydia*-infecting bacteriophage found in diverse bacterial species have been reported to occur in the genomes of two multicellular eukaryotes^61^. The presence of prokaryotic sequences in tumor cells have also been shown^62^. On the other hand, genes with eukaryotic homology are present in phage genomes, which suggests that horizontal gene transfer may happen in both directions^16^. Notably, several phages have gene delivery tools that facilitate intracellular movement of phage DNA through the nuclear envelope^17^. The proteins responsible for this are covalently linked to the phage genome ends and contain eukaryotic nuclear localization signals that promote their accumulation in the nucleus. Once inside the eukaryotic cell, these terminal proteins provide mechanism for localizing foreign DNA for gene expression or even integration into the cell genome^17, 63^. Our pulse-chase experiment showed that the internalized phages could be recovered from the cells in their infective form up to 24 h, followed by an effective inactivation of phages within the cells. The penetration of the phage genome through the cell membrane barrier at the outer border of the cell is a prerequisite to any incidental gene exchange processes within the cell, the detailed mechanisms of which remain to be explored.

The close physical coexistence of phages and eukaryotes may offer many opportunities for gene exchanges to occur. Considering the tremendous diversity of genotypic and phenotypic phage variants in nature and the rapid evolutionary capacity of the phage genomes, it is not unlikely that over a long evolutionary time period phages have influenced the eukaryotic genomes in multiple ways. The mechanism of crossing the cell membrane barrier between the bacterial and eukaryotic gene pools may be one contributing factor to enable this influence to occur.

## Methods

### Cell culture

The cell lines obtained from the American Type Culture Collection (ATCC) included human neuroblastoma cell lines SK-N-SH (ATCC HTB-11), SK-N-AS (ATCC CRL-2137) and SH-SY5Y (ATCC CRL-2266), human foreskin fibroblast cell line BJ (ATCC CRL-2522), and baby hamster kidney fibroblast cell line BHK-21 (ATCC CCL-10). The polysialic acid-expressing cell line kSK-N-SH was previously established in our laboratory from SK-N-SH cells by immunomagnetic bead separation^31^, and was authenticated together with SK-N-SH cell line by using GenePrint® 10 system (Institute for Molecular Medicine Finland). Cell lines were checked for the absence of mycoplasma infections using the EZ-PCR Mycoplasma Test Kit (Biological Industries). All cells were cultured in DMEM containing 4.5 g l^−1^ glucose (Sigma-Aldrich), supplemented with 10% fetal bovine serum (HyClone), and 100 U ml^−1^ penicillin and 100 μg ml^−1^ streptomycin (Gibco), and maintained at 37 °C in 5% CO_2_ under a humidified atmosphere. For binding and uptake assays, the cells were grown on 13-mm glass slides in 24-well plates to approximately 70% confluence.

### Propagation and purification of bacteriophages

The *E. coli* bacteriophages PK1A and PK1A2 have been described by Gross et al.^64^ and Pelkonen et al.^22^, respectively. Preliminary sequence analysis reveals that PK1A2 is very closely related to *E. coli* K1-specific phage K1E, as 94% of the genome is 96% identical to K1E (the genome sequence was deposited in GenBank, accession number MG004687). Thus, the phage belongs to the SP6 subgroup within the T7 supergroup^65^. The K1-encapsulated *E. coli* host strain IH3088 was used for the propagation of PK1A, and strain EH954 derived from IH3088 that has a highly reduced amount of the K1 capsule for PK1A2^66^. The phages were purified as described before^67^. Briefly, lysates of phages were obtained by infecting exponentially growing host cells in Luria Bertani (LB) broth at 37 °C. After removing the bacterial debris by centrifugation, the phages were precipitated at 4 °C by addition of 10% polyethylene glycol 6000, 0.5 M NaCl, followed by centrifugation. Further purification and concentration of the phages were achieved by CsCl density gradient ultracentrifugation. Finally, the phages collected from the gradient were dialyzed against 25 mM sodium phosphate, pH 7.4, 150 mM NaCl at 4 °C, and the titer (plaque-forming units per ml, p.f.u. ml^−1^) was determined by plaque assay.

### Labeling of phage capsid

To label the phage capsid with amine-reactive FITC, the buffer of purified phages (1 × 10^11^ p.f.u.) was changed using Amicon Ultra-15 centrifugal filter devices (Millipore) to 0.1 M carbonate buffer pH 9.0, and FITC (Sigma-Aldrich) was added to a final concentration of 0.25 mg ml^−1^. After incubation for 1 h at room temperature with rotation in the dark, unbound dye was removed via buffer exchange into 25 mM sodium phosphate, pH 7.4, 150 mM NaCl using centrifugal filter devices. To label with amine-reactive DSB-X biotin, the DSB-X Biotin Protein Labeling Kit (Molecular Probes) was used according to the manufacturer’s instructions. Briefly, phages (4 × 10^10^ p.f.u.) were resuspended in 220 µl of 0.1 M carbonate buffer, pH 8.5, and DSB-X biotin was added to a final concentration of 0.09 mg ml^−1^. After incubation for 1.5 h at room temperature with stirring, excess biotin was removed by purification resin.

### Labeling of phage DNA

To incorporate nucleoside analog EdU or BrdU into phage genome, phages were propagated in host bacteria at 37 °C in presence of 50 μM EdU (Molecular Probes) or 300 µM BrdU (Roche), added at the beginning of the infection, and purified by CsCl density gradient ultracentrifugation. The incorporations were tested on immobilized phage particles. The labeled phages or unlabeled control phages (5 × 10^5^ p.f.u. in a volume of 5 µl) in phosphate-buffered saline, pH 7.4 (PBS, Gibco) were spotted onto Polysine adhesion slides (Thermo Scientific) and air-dried at 37 °C for 10 min. To open phage capsids and make the incorporated nucleoside analogs EdU or BrdU accessible for the staining reagents, the samples were pretreated with a 4 M HCl for 20 min at room temperature and then washed six times with PBS. To detect exposure of the EdU or BrdU-labeled DNA of the phage particles, the stainings were also performed without HCl pretreatment. For the detection of EdU, the Click-iT Plus EdU Alexa Fluor 488 Imaging Kit (Molecular Probes) was used according to the manufacturer’s instructions. For the detection of BrdU, mouse anti-BrdU antibody (clone B44, BD Biosciences) diluted 1:50 in 1% BSA in PBS was used, followed by Alexa Fluor 555 Plus goat anti-mouse antibody (A32727, Molecular Probes) diluted 1:500 in 1% BSA in PBS, each for 1 h at room temperature. For mounting, 5 µl of antifade solution (0.5% ascorbic acid, 50% glycerol in PBS) was added to center of sample and covered with a coverslip. Samples were examined by an Olympus BX50F-3 microscope and imaged by a Retiga 6000 CCD camera and Image-Pro Plus 7.0 software.

### Construction and purification of recombinant proteins

The pQE31-based constructs pFEndoNA2 and pFEndoNA for inactive and active endosialidase-GFP fusion proteins, respectively, were available from previous work^68^. For DsRed fusion derivative, the *gfp* gene in pFEndoNA2 was replaced with NotI and SpeI restriction sites and a PCR-amplified DsRed-Monomer coding sequence (Clontech). Briefly, linear pFEndoNA2 without the *gfp* gene was amplified using inverse PCR with Phusion High-Fidelity DNA Polymerase (Thermo Scientific) and 5% DMSO as a PCR additive as recommended by the manufacturer. The purified PCR product was treated with DpnI in order to digest the parental plasmid strands and ligated with the DsRed-Monomer coding sequence, yielding the plasmid pDsEndoNA2. The construct was verified by DNA sequencing at the Institute of Biotechnology at the University of Helsinki.

Constructs were expressed in *E. coli* M15 (pREP4) expression strain as N-terminal histidine-tagged fusion proteins and purified under native conditions using nickel-nitrilotriacetic acid resin (Qiagen) as described before^68^.

### Phage binding to cells and immunofluorescent staining

Before phage binding and immunostaining, the eukaryotic cells were washed with PBS and fixed with cold 4% paraformaldehyde in PBS for 25 min at room temperature. After washing three times with PBS, non-specific binding was blocked with 1.5% normal horse serum (Vector Laboratories) in PBS for 1 h at room temperature. FITC-labeled phages (2.5 × 10^8^ p.f.u.) in PBS were added onto the fixed cells and incubated for 1 h at room temperature. A control experiment was performed in the presence of unlabeled phages, PBS without phages or PBS with a free FITC label. As further control, the FITC-labeled phages were incubated with cells in the presence of free bacterial polysialic acid (1 mg ml^−1^ colominic acid, poly-2,8-N-acetylneuraminic acid sodium salt, Sigma-Aldrich), which is structurally identical to eukaryotic cell surface polysialic acid^69^. For enzymatic removal of cell surface polysialic acid before phage binding^67^, cells were treated with 10 μg ml^−1^ of active endosialidase-GFP fusion protein for 3 h at 37 °C in a 5% CO_2_ humidified atmosphere, followed by two washes with PBS and fixation in paraformaldehyde in PBS.

For detection of polysialic acid expression, cells were stained with 10 µg ml^−1^ inactive endosialidase-GFP fusion protein or inactive endosialidase-DsRed fusion protein in PBS for 1 h at room temperature. For the staining of the protein carrier of polysialic acid, NCAM, rabbit polyclonal anti-human NCAM antibody (AB5032, Millipore) diluted 1:250 in PBS was used, followed by Alexa Fluor 555 goat anti-rabbit secondary antibody (A-21429, Molecular Probes) diluted 1:500 in PBS, each for 2 h at room temperature. Secondary antibody control omitting the NCAM antibody was prepared for each tested cell line and confirmed the specificity of the staining. The coverslips were washed three times with PBS, mounted with ProLong Mounting Medium with DAPI (Molecular Probes) and visualized with fluorescence microscopy. Polysialic acid expression and NCAM staining were confirmed by flow cytometry as described previously^31^.

### Phage internalization assay and immunofluorescence

For analysis of phage internalization in mammalian cells, cells were incubated with FITC-labeled phages (2.5 × 10^8^ p.f.u.) in cell culture medium at 37 °C in a 5% CO_2_ humidified atmosphere. After various time intervals, the cells were washed with PBS and fixed with cold 4% paraformaldehyde in PBS for 25 min at room temperature. Finally, the coverslips were washed, mounted and observed by fluorescence microscopy.

For the staining of biotin-labeled phage, cells grown on coverslips were incubated with the phages (2.5 × 10^8^ p.f.u.) for 24 h at 37 °C in a 5% CO_2_ humidified atmosphere. The cells were washed with PBS, fixed with cold 4% paraformaldehyde in PBS for 25 min, and permeabilized by incubation for 5 min at room temperature with 0.2% Triton X-100 in PBS. After washing three times with PBS and incubation with 1.5% normal horse serum in PBS for 1 h at room temperature, the cells were stained for 1 h at room temperature with Alexa Fluor 488 FluoroNanogold-Streptavidin (Nanoprobes) probe diluted 1:100 in PBS. In parallel, non-permeabilized control cells were stained with the probe without Triton X-100 treatment.

For the staining of EdU-labeled phage, cells incubated with the phages (2.5 × 10^8^ p.f.u.) for various intervals were fixed, washed, permeabilized and visualized by the Click-iT Plus EdU Alexa Fluor 488 Imaging Kit (Molecular Probes) according to the manufacturer’s protocol. For the staining of BrdU-labeled phage, cell samples fixed with paraformaldehyde were permeabilized with 0.2% Triton X-100 in PBS for 10 min at room temperature and then immunolabelling was performed as described above for immobilized phage particles. For HCl pretreatment samples, the coverslips were incubated with 4 M HCl for 20 min at room temperature and then washed six times with PBS after the permeabilization step. Finally, coverslips were incubated with 20 ng ml^−^^1^ DAPI (Sigma-Aldrich) for 5 min at room temperature and washed three times with PBS, followed by mounting in antifade solution (0.5% ascorbic acid, 50% glycerol in PBS), sealing with nail polish and examination under fluorescence microscope. For the quantification of EdU-positive vesicles, the dye positive vesicles from at least 100 different cells were counted for each individual experiment.

For EEA1 or LAMP1 immunolabelling, cells incubated with FITC-labeled phages were fixed with paraformaldehyde and permeabilized with 0.1% Triton X-100, 1.5% normal horse serum in PBS for 1 h at room temperature. Then the cells were incubated at 4 °C overnight with rabbit monoclonal EEA1 (C45B10, Cell Signaling) or LAMP1 (D2D11, Cell Signaling) antibody diluted 1:200. The secondary antibody Alexa Fluor 555 goat anti-rabbit (A-21429, Molecular Probes) was used at 1:500 for 2 h at room temperature. For cells incubated with EdU-labeled phages, the antibody incubations were performed after EdU detection. To stain lysosomes, live cells were incubated with 50 nM LysoTracker Red DND-99 (Molecular Probes) in cell culture medium for 30 min at 37 °C in a 5% CO_2_ humidified atmosphere. For the quantification of percent overlap between phage clusters and endocytic markers, phage-positive vesicles were identified and scored whether a marker of interest was also present on the vesicles. A minimum of 300 vesicles from at least ten different cells was quantified for each individual experiment.

### Fluorescence in situ hybridization

Alexa Fluor 488-labeled DNA probes were generated by nick translation using fluorescence in situ hybridization (FISH) Tag DNA Multicolor Kit (Molecular Probes) and PK1A2 genomic DNA as the template according to the manufacturer’s instructions. The method aims at the generation of a whole-genome mixture of overlapping DNA fragments. The average length of ~500 bp for optimal hybridization was verified by agarose gel electrophoresis.

To detect phage genomes, cells incubated with the phages (2.5 × 10^8^ p.f.u.) and fixed with cold 4% paraformaldehyde in PBS for 25 min at room temperature were permeabilized with 0.5% Triton X-100 in PBS for 10 min at room temperature and washed three times with PBS. Coverslips were inverted over 25 μl of hybridization buffer made of 15% ethylene carbonate, 20% dextran sulfate, 600 mM NaCl, 30 mM sodium citrate pH 7.0^70^ and incubated for 10 min at 80 °C. Coverslips were then placed on top of 5 µl of hybridization buffer that contained 6 ng µl^-1^ labeled probes and was denaturated for 10 min at 80 °C. Samples were incubated for 2.5 h at 45 °C in a humid chamber, followed by three washes with 15% ethylene carbonate, 2 × SSC (300 mM NaCl, 30 mM sodium citrate pH 7.0) for 5 min each at 45 °C and three washes in 2 × SSC at room temperature. Coverslips were incubated with 20 ng ml^-1^ DAPI (Sigma-Aldrich) for 5 min at room temperature and washed three times with PBS. Finally, coverslips were mounted in SlowFade Gold antifade reagent (Molecular Probes) and examined under fluorescence microscope.

### Proteolytic inactivation of phage

Stability of the phage toward proteolytic inactivation was tested by treating phages (2.5 × 10^8^ p.f.u.) with a solution containing 2.5 mg ml^−1^ trypsin-EDTA (Gibco) for 5 min at 37 °C, 3 or 30 mg ml^−1^ subtilisin (Sigma-Aldrich) in 50 mM Tris-HCl, 150 mM NaCl, pH 7.5 for 1 h at room temperature, or 2 or 20 mg ml^−1^ proteinase K (Sigma-Aldrich) in PBS for 1 h at 37 °C. After incubation, the proteases were inactivated by the addition of cOmplete EDTA-free protease inhibitor cocktail (Roche) according to the manufacturer’s instructions and the samples were assayed for p.f.u. by titering.

### Removal of cell surface phage

For removing cell membrane-bound phages, both acidic and polysialic acid buffer conditions were tested. For acid wash, the cells were prewashed with ice-cold PBS and then incubated once or twice with 0.2 M glycine-HCl, pH 2.2 for 5 min on ice, followed by two washes with ice-cold PBS. For polysialic acid treatment, the cells were prewashed twice with cell culture medium and then incubated three times with 1 mg ml^−1^ free bacterial polysialic acid in cell culture medium for 5 min at 37 °C in a 5% CO_2_ humidified atmosphere, followed by five washes with PBS.

### Quantification of phages

To assess the amount of internalized phages, the cells grown in 24-well plates to approximately 70% confluence were incubated with phages (2.5 × 10^8^ p.f.u.) for various time intervals at 37 °C in a 5% CO_2_ humidified atmosphere. The cells were then polysialic acid-treated in the plates as described above. Then the cells were detached with a brief trypsinization, transferred into microcentrifuge tubes and pelleted for 4 min at 400 g. After washing twice with PBS, cells were lysed according to the procedure of Ivanenkov et al.^34^ Briefly, the cells were resuspended in 0.5 ml of lysis buffer (2% sodium deoxycholate, 10 mM Tris-HCl, 2 mM EDTA, pH 8.0), vortexed for 10 s and incubated for 1 h at room temperature. The lysis buffer had no effect on phage infectivity after 1 h incubation. After cell lysis, the lysates were vortexed for 10 s and p.f.u. were quantified by titering. To prevent counting of any extracellular phages that could survive polysialic acid treatment procedure, samples without the cell lysis step were made in parallel with each assay. The titer of internalized phages was calculated by subtracting the p.f.u. value obtained with non-lysed cells from p.f.u. value obtained with lysed cells.

For quantification of cell surface bound phages, the cells incubated with phages were directly washed three times with PBS without polysialic acid treatment. Cells were then detached and washed as described above. The lysis step was omitted and phages were quantified by titering.

For pulse-chase assay, the cells were first incubated with pulse medium containing 2.5 × 10^8^ p.f.u. of phage for 24 h at 37 °C in a 5% CO_2_ humidified atmosphere. The pulse medium was aspirated, and extracellular phages were removed by polysialic acid treatment procedure as described above. Cell culture medium was then added to cells followed by incubation for 24 or 48 h at 37 °C in a 5% CO2 humidified atmosphere. Following the chase, internalized and cell surface bound phages were quantified as described above.

### Cell viability assay

The kSK-N-SH cells (5 × 10^4^ cells per well) were seeded in 96-well plates. After overnight attachment, the cells were incubated with increasing amounts of the phages PK1A2 or PK1A (10^3^–10^9^ p.f.u. per well) for 24 h at 37 °C in a 5% CO_2_ humidified atmosphere. A 3-(4,5-dimetrylthiazol-2-yl)-2,5-diphenyltetrazolium bromide (MTT, Sigma-Aldrich) stock solution (5 mg ml^−1^ in RPMI 1640 without phenol red, Gibco) was filter sterilized and added to the final concentration of 0.5 mg ml^−1^, followed by incubation for a further 3 h. Then the medium was aspirated and 100 µl of 4 mM HCl in anhydrous isopropanol was added to each wells. After incubating for an additional 30 min at room temperature in the dark, the absorbance was measured with an ELISA recorder (Multiskan GO, Thermo Scientific) at a wavelength of 560 nm with background subtraction at 690 nm. Cell viability was calculated in relation to control (non-treated) cells.

### Transmission electron microscopy

The cells grown on 13-mm glass slides in 24-well plates were incubated with phages (2.5 × 10^8^ p.f.u.) for various time intervals at 37 °C in a 5% CO_2_ humidified atmosphere. After washing with PBS, cells were fixed with freshly made 4% paraformaldehyde in PBS for 30 min at room temperature and then washed three times with PBS. For pre-embedding labeling of biotin-phages, the cells were permeabilized and blocked with saponin buffer (0.01% saponin, 0.1% bovine serum albumin in 100 mM sodium phosphate buffer, pH 7.4) for 8 min and then reacted for 1 h at room temperature with Alexa Fluor 488 FluoroNanogold-Streptavidin (Nanoprobes) probe diluted 1:200 in saponin buffer. After washing three times with saponin buffer and 100 mM sodium phosphate buffer, pH 7.4, the cells were post-fixed with 1% glutaraldehyde in 100 mM sodium phosphate buffer for 10 min and quenched with 50 mM glycine (Sigma-Aldrich) in 100 mM sodium phosphate buffer for 5 min at room temperature. Following washes with double-distilled water, gold particles were silver-intensified using the HQ Silver Enhancement Kit (Nanoprobes) for about 5 min in the dark according to manufacturer’s directions, followed by gold toning (three washes with 2% sodium acetate, toning in 0.05% gold chloride on ice, and excess silver removed with 0.3% sodium thiosulfate). The cells were further post-fixed in 1% reduced osmium tetroxide, 15 mg ml^−1^ potassium hexacyanoferrate, 100 mM sodium cacodylate buffer, pH 7.4 for 1 h at 4 °C, dehydrated with 70%, 96% and absolute ethanol, and flat embedded in Epon (TAAB 812) for 2 h prior to polymerization for 14 h at 60 °C. Ultrathin sections were cut parallel to the coverslip and collected on copper mesh grids, followed by post-staining with uranyl acetate and lead citrate. Sample grids were observed using a JEM-1400 transmission electron microscope (JEOL) operating at 120 kV and equipped with an ORIUS SC1000 bottom mounted CCD camera (Gatan).

### Statistical analysis

For quantitative data, results were reported as the mean ± s.d. For phage quantification assays, differences between mean values were tested for significance by performing an unpaired, two-sided Student’s *t*-test. *P-*values of <0.05 were considered statistically significant.

### Data availability

Phage PK1A2 genome sequence data have been deposited in the GenBank nucleotide database under accession number MG004687. All other relevant data supporting the findings of this study are available within the article and its Supplementary Information files, or from the corresponding author on request.

## Electronic supplementary material

Peer Review File

Supplementary Information
