# Supplementary material for: Internalization of a polysialic acid-binding Escherichia coli bacteriophage into eukaryotic neuroblastoma cells
Source: Nat Commun. 2017 Dec 4;8:1915. doi: 10.1038/s41467-017-02057-3 (PMC5715158; doi:10.1038/s41467-017-02057-3)
Supplement: Supplementary file 2 — Supplementary Information [file 41467_2017_2057_MOESM2_ESM.pdf]

Supplementary Figures

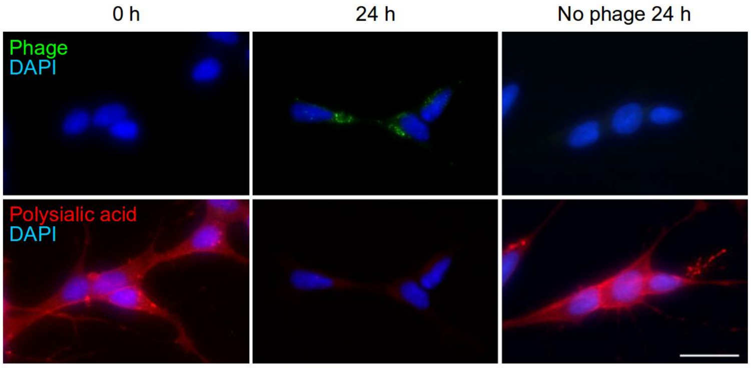

Supplementary Figure 1. Disappearance of polysialic acid from the cell surface is dependent on phage internalization. Live kSK-N-SH cells were incubated with or without FITC-labelled PK1A2 phages (green) at 37 °C for the times indicated. After incubation, the cells were fixed and stained for surface-expressed polysialic acid (red). Nuclei were stained with DAPI (blue). Representative images from three biological replicates are shown. The scale bars represent 20 µm.

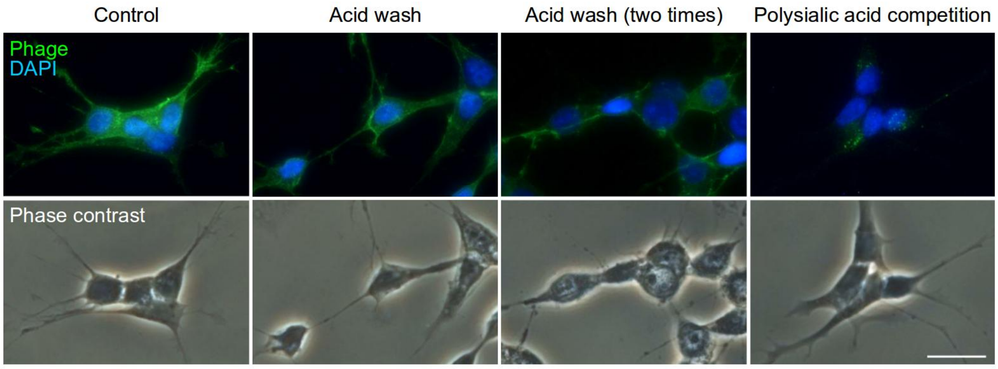

Supplementary Figure 2. Effect of acid wash or polysialic acid competition on removal of non-internalized phages from the cell surface. Live kSK-N-SH cells were incubated with FITC-labelled PK1A2 phages (green) for 1 h at 37 °C and then acid-washed or polysialic acid-treated. Nuclei were stained with DAPI (blue). Representative images from three biological replicates are shown. The scale bar represents 20 µm.

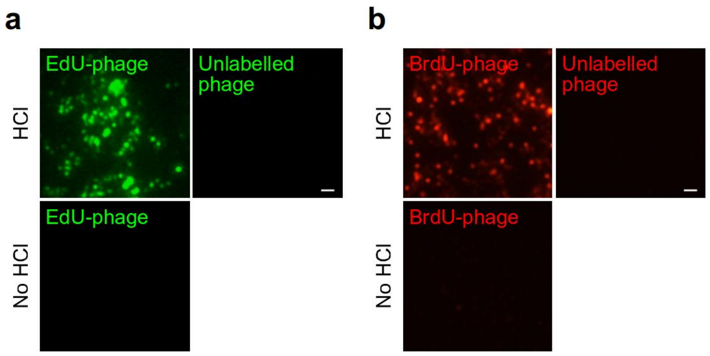

Supplementary Figure 3. Incorporation of the nucleoside analogs BrdU and EdU into bacteriophage. (a, b) Fluorescence microscopic images of DNA-labelled PK1A2 phages attached to polylysine-coated glass slides and stained for EdU (a) or BrdU (b) with or without HCl pretreatment. Unlabelled phage particles were used as control of staining specificity. The scale bars represent 1 µm.

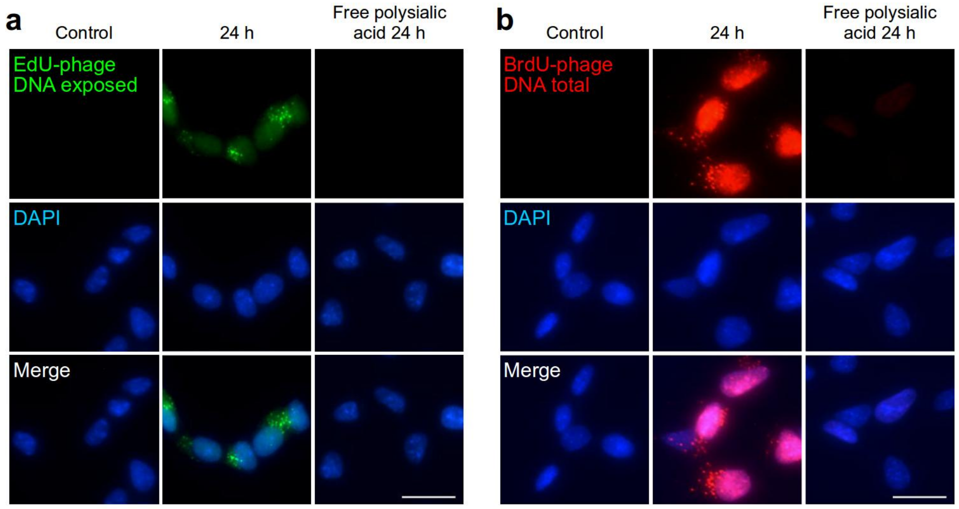

Supplementary Figure 4. Nuclear association of phage-derived nucleosides is dependent on phage internalization. kSK-N-SH cells were incubated with EdU (a) or BrdU (b) labelled PK1A2 phages (green and red, respectively) in the absence or the presence of free polysialic acid for 24 h at 37 °C. Labelled phages were detected under the conditions showing exposed DNA or total DNA as described in Methods. Nuclei were stained with DAPI (blue). Representative images from three biological replicates are shown. The scale bars represent 20 µm.

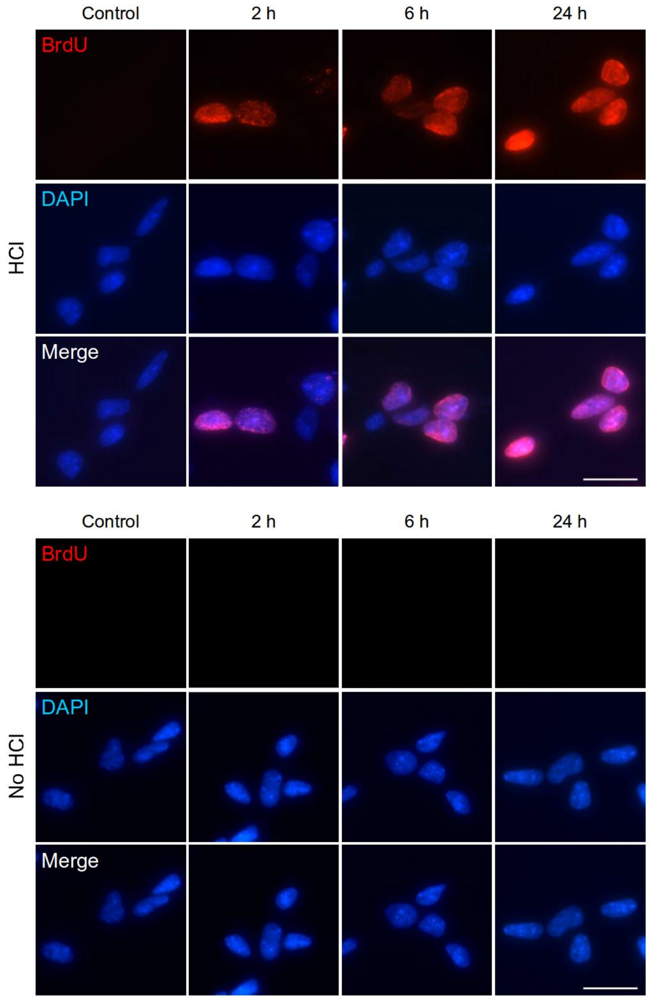

Supplementary Figure 5. Detection of BrdU incorporated into the nuclear DNA of cultured kSK-N-SH cells. The cells cultured in the presence of 10 μM BrdU for 2, 6 or 24 h were immunostained for BrdU with (upper panel) or without HCl pretreatment (lower panel) to total DNA or exposed single-stranded DNA, respectively. Cells without added BrdU were used as control of staining specificity. Nuclei were stained with DAPI (blue). Representative images from three biological replicates are shown. The scale bars represent 20 μm.
