# Supplementary material for: Internalization of a polysialic acid-binding Escherichia coli bacteriophage into eukaryotic neuroblastoma cells
Source: Nat Commun. 2017 Dec 4;8:1915. doi: 10.1038/s41467-017-02057-3 (PMC5715158; doi:10.1038/s41467-017-02057-3)
Supplement: Supplementary file 1 — Peer Review File [file 41467_2017_2057_MOESM1_ESM.pdf]

Reviewers' comments:

Reviewer #1 (Remarks to the Author):

Lehti et al convincingly showed that the E. coli podophage PK1A2 is capable of penetration into human neuroblastoma cell line expressing polysialic acid at its surface and stay infectious for at least 24 hours.

Specific comments

1. A line numbering system in the manuscript would have been appreciated. I created one to ease my review.

2. The title is too general and does not precisely reflect the study.

3. Abstract: The abstract is remarkably vague. The name of the phage should be added, its host bacterium, the cell surface polysaccharide should be indicated, the cell line should be added, as well as the techniques used to show penetration of phages in eukaryotic cells.

4. P3L15 / P9 L1: you wrote: “There is currently no experimental evidence indicating that phages are able to traverse the eukaryotic membrane and enter the cells”. This sentence needs to be either tone down or better explained. It is my understanding that over 40 years it was shown that phage lambda is capable of transducing mammalian cells (Geier and Merril, 1972; Merril, Geier, and Petricciani, 1971) both in vitro and in vivo and and they may be taken up by nonphagocytic mechanisms (Merril et al. 1971). Please clarify your sentence.

5. P3 L15: One can easily envision that a phage-infected bacterium can be taken up (phagocytosis for example) by cells (dendritic, macrophages, etc).

6. P3 L18: But it does not provide entry to the nucleus.

7. P4 L4: You should indicate here that this phage belongs to the Podoviridae family, which is different than lambda. Anything known about its genome?

8. P4L4: You should indicate here that PK1A2 differs by two amino acids in the tailspike protein endosialidase that abolish catalytic activity but still permit polysialic acid binding. Is it common that phages bind to polysialic acid?

9. P4 L5: Perhaps it should be indicated that the capsular polysialic acid, or K1 antigen, is a linear homopolymer of α2,8-linked N-acetylneuraminic acid residues and plays an essential role in pathogenesis by protecting the invasive bacteria from host innate immunity (Deszo et al. 2005).

10. P4 L7: It seems that this has been done previously. See Aalto et al. Mutant bacteriophage with non-catalytic endosialidase binds to both bacterial and eukaryotic polysialic acid and can be used as probe for its detection. Glycoconjugate J. 18, 751-758 (2001). Clarify.

11. P4 L8: Polysialic acid is naturally found in which types of human cells?

12. P4 L12: Perhaps some additional info could be provided on the cell line, especially for those that are not familiar with neuroblastoma cell line.

13. P4 L25: It should be indicated that this is bacterial polysialic acid. Is the phage receptor found in this prep? Could this explain the dissociation and the high-affinity reaction with the bacterial polysialic acid? The excess was determined how?

14. P5 L1: Phage PK1A has the enzyme endosialidase and thus should still bind the polysialic acid (as indicated by Jakobsson et al. 2007). It seems that a one-hour incubation (at which temperature?) is enough for the phages to bind, hydrolyse the polysialic acid and be released. Perhaps testing another unrelated phage (as a negative control) without polysialic acid-binding capacity would be better?

15. P5 L10/L22: After 6 hours, cell surface polysialic acid had disappeared? Why? This should be discussed.

16. P5 L14: Phages (plural). Check throughout the manuscript.

17. P6 L3: Bacterial polysialic acid

18. P6 L7: Phages are not viable per se. Consider revising. Check throughout the manuscript.

19. P6 L8: Any reason why the proteolytic treatments were not effective? Have you tried to increase the enzyme concentrations? This should be discussed.

20. P6 L25: Why K1? According to P11 L20, the host of phage PK1A2 is strain EH954?

21. P7 L3: 30%? Compared to the titer at 2h?

22. P7 L14: Considering that in the introduction you are mentioning DNA transfer between phage and human cells, I am surprised that the presence of phage DNA was not tested at 48 hours.

23. P7 L21: Consider replacing head by capsid.

24. P8 L5: Is it surprising to find that phages appeared to accumulate into large vesicles over time? This should be discussed.

25. P10 L11: Is there such protein in phage PK1A2 (proteins covalently linked to the genome ends and contain eukaryotic nuclear localization signals that promote their accumulation in the nucleus)? If not, what is the likelihood of reaching the nucleus?

26. P11 L24: Is there any polysialic acid left in the phage prep?

27. P13 L9-12: Phage PK1A seems more efficient than the purified protein in hydrolyzing polysialic acid! This should be discussed.

28. Fig 1: Phages were incubated for how long?

29. Fig 3e: Why not test the control PK1A phages containing catalytically active endosialidase at 4°C?

Reviewer #2 (Remarks to the Author):

This is a potentially interesting study that suggests that phage might be capable of mediating the horizontal transfer of DNA between prokaryotes and eukaryotes. Given the abundance of phage in our world and the fact that all metazoan systems appear to exist in close harmony with their own unique microbiome and associate phages, phage have ample opportunity to interact with eukaryotic cells. Yet there is very little if any reliable data indicating that they can transfer genetic material to eukaryotic cells. In this study the authors show that molecules very similar to bacterial

phage receptors are expressed on mammalian cells and that these molecules can indeed bind phage particles and apparently cause them to be internalised into cells. It seems that the internalised phage enter intracellular vesicles but, over a period of 1 to 2 days, loose their integrity, as measured by their infectivity on cognate bacterial hosts. These are interesting observations that the authors suggest establishes a case for phage being able to "cross the prokaryote–eukaryote barrier", but in my view the study lacks key details that would enable the authors to make this claim with confidence.

The authors suggest that phage particles are taken up by receptor-mediated endocytosis, which may well be true. But if this is the case it would be relatively easy to identify the compartments in which internalised the particles are located by using well characterised markers for endocytic organelles and immunofluorescence analysis. Although the authors show phage uptake, crucially they do not show transfer of phage DNA to cells. Although phage appear to be taken up into vesicles, they are still separated from the cytoplasm by a vesicle membrane, so the notion that they reach the 'cell interior' (line 184) is not really established. Moreover, as the internalised phage gradually loose integrity, is it not just as likely they are degraded in lysosomes? For this study to really make an impact it will be crucial to show that phage DNA is delivered into the cell cytoplasm or nucleus.

Minor points:-

Line 83: Epifluorescence imaging lacks the resolution necessary for the authors to claim that receptors and phage are 'co-localised'. They should say 'have similar distributions'.

Line 93: Why do the authors say 'apparently due to their polysialic acid cleaving activity', has this been established?

Line 99: From this data the authors can't say whether the phage are in the cytoplasm or within vesicles. All that they can say is that they appear to have been internalised.

Fig 6a: The right hand 24 hr panel should be removed. It's impossible to say whether the vacuole contains phage particles or is a multivesicular body.

Reviewer #3 (Remarks to the Author):

The article by Jukka Finne and collaborators provide solid evidences of the entrance of PK1A2 phage into polysialic acid-containing human cells. The experiments are clear and I do believe that all negative and positive controls demonstrate the specific binding and internalization of the phage intro the mammalian cells. This novel piece of data further supports previous hypotheses about the role of bacteriophages as vectors for prokaryotes-to-eukaryotes horizontal gene transfer, a phenomenon that have been somewhat controversial. Therefore, I am sure that the paper will raise the interest of researchers from both prokaryotic and eukaryotic side as well as evolutionary and computational biologist.
I have some comments that hopefully may help to strength the manuscript and clarify some aspects of the Discussion regarding the biological importance of these findings.
1. The authors showed that phage infectivity drops after 48 h inside the eukaryotic endocytic pathway, which would be consequence of the destabilization of the viral particles. It would be very interesting to examine this more deeply. In particular, I wonder whether this virions deactivation eventually leads to viral DNA partial leakage or release, which would be required to continue its journey to the cell nucleus.
2. Regarding the discussion of the biological relevance of bacterial viruses inside eukaryotic cells, I agree that HGT is a very likely hypothesis and it is well presented in the discussion. However, once

phage DNA eventually reach the cell nucleus, either in a somatic or non-somatic cell, other effects with biological meaning may occur and should be considered in the Discussion section. For instance, modulation of immune system by phage genes has been already suggested (ref. 45). The presence of prokaryotic sequences in tumor cells have been also showed (Riley D.R. et al. Plos Comput Biol; 9(6) 2013).

3. Finally, as the authors mentioned, the nuclear envelope constitutes an additional barrier that only very small DNA molecules can overcome. Phage terminal proteins have been shown to enhance gene delivery by means of functional NLSs. However, although widespread among different viral families and other diverse replicons, protein-primed DNA replication is not a very frequent mechanism. Interestingly, other phage proteins have been shown to contain functional NLSs, like P1 Cre recombinase and other DNA binding proteins, that may also favor nuclear internalization of phage DNA. I wonder if there may be putative NLSs-containing proteins among the PK1A or PK1A2 proteome.

Other minor points:

1. Lane 59. This statement seems to me somewhat misleading. As the authors mentioned in the immediately above sentence (lane 57), phage genes are functional in eukaryotic cells, which is a strong “experimental evidence” that phages (at least in those cases) indeed entered the eukaryotic cell. I believe that the authors should highlight the lack of a direct evidence that show the detailed mechanism of virion binding and subsequent internalization in a nonphagocytic cell, as has been demonstrated in this work.

2. Lane 236. “Infected cells” is not formally correct, since the phage are not replicating within the eukaryotic cells. One might say abortive infection, but I believe that it would be better to avoid this term.

Responses to reviewers

We thank the editor and all the reviewers for their careful reading and valuable comments. We have performed several novel experiments to address the reviewer's suggestions. Overall, the constructive comments helped to improve the study, and we think that the additional data strengthen our observations and conclusions significantly. We hope that the improved version of the manuscript is now suitable for publication in Nature Communications.

The changes made are indicated below and highlighted in the revised manuscript.

Reviewer #1 (Remarks to the Author):

Lehti et al convincingly showed that the E. coli podophage PK1A2 is capable of penetration into human neuroblastoma cell line expressing polysialic acid at its surface and stay infectious for at least 24 hours.

Specific comments

1. A line numbering system in the manuscript would have been appreciated. I created one to ease my review.

> Response: We apologize for the inconvenience related to line numbers. They should have been included by the online submission system during the PDF conversion. In the revised manuscript, we have included line numbers within the text.

2. The title is too general and does not precisely reflect the study.

> Response: We thank the Reviewer for advice and have modified the title to include more details (lines 4-5).

3. Abstract: The abstract is remarkably vague. The name of the phage should be added, its host bacterium, the cell surface polysaccharide should be indicated, the cell line should be added, as well as the techniques used to show penetration of phages in eukaryotic cells.

> Response: As suggested by the reviewer, we have revised the abstract to include the requested details (lines 20-29).

4. P3L15 / P9 L1: you wrote: "There is currently no experimental evidence indicating that phages are able to traverse the eukaryotic membrane and enter the cells". This sentence needs to be either tone down or better explained. It is my understanding that over 40 years it was shown that phage lambda is capable of transducing mammalian cells (Geier and Merril, 1972; Merril, Geier, and Petricciani, 1971) both in vitro and in vivo and and they may be taken up by nonphagocytic mechanisms (Merril et al. 1971). Please clarify your sentence.

Response: We thank the reviewer for bringing up this valuable point. We have rephrased the corresponding sentences in the Introduction and Discussion sections to take into account the mentioned studies (lines 58-60, 250-252).

5. P3 L15: One can easily envision that a phage-infected bacterium can be taken up (phagocytosis for example) by cells (dendritic, macrophages, etc).

Response: We agree and have now indicated both in Introduction and Discussion that we are dealing with nonphagocytic cells (lines 60, 252).

6. P3 L18: But it does not provide entry to the nucleus.

Response: True, the phage internalization is not a direct evidence to claim the entry of phage genetic material inside the nucleus. Nuclear entry is presumably a rare event which is not detected under the time course and conditions of the experiments. We have now added a note in the Discussion to point it out (lines 263-265).

7. P4 L4: You should indicate here that this phage belongs to the Podoviridae family, which is different than lambda. Anything known about its genome?

Response: We have now indicated the family for PK1A2 phage in the text (line 70). We also sequenced both PK1A2 and PK1A genomes. The genomes consist of a double-stranded linear DNA molecule of 44 498 bp and preliminary sequence analysis revealed that the phages are very closely related to *E. coli* K1-specific phage K1E, as 94% of the PK1A2 or PK1A genome is 96% identical to K1E. Thus the phages belong to the SP6 subgroup within the T7 supergroup. This information has now been added to the Methods section (lines 329-331).

The questions of terminal proteins and nuclear localization signals are discussed under point 25.

8. P4L4: You should indicate here that PK1A2 differs by two amino acids in the tailspike protein endosialidase that abolish catalytic activity but still permit polysialic acid binding. Is it common that phages bind to polysialic acid?

Response: As suggested by the reviewer, we have now added the information of amino acid substitutions to the beginning of revised Results (lines 74-76).

So far, around 30 enterobacterial phages that target specifically the *E. coli* K1 polysialic acid capsule have been isolated (Jakobsson et al., 2015). However, to our knowledge, polysialic acid-specific phages for other bacterial hosts have not been characterized.

9. P4 L5: Perhaps it should be indicated that the capsular polysialic acid, or K1 antigen, is a linear homopolymer

of α2,8-linked N-acetylneuraminic acid residues and plays an essential role in pathogenesis by protecting the invasive bacteria from host innate immunity (Deszo et al. 2005).

Response: As instructed, we have now indicated the composition of K1 antigen as well as its protective role against host immune responses in the text (lines 72, 73-74).

10. P4 L7: It seems that this has been done previously. See Aalto et al. Mutant bacteriophage with non-catalytic endosialidase binds to both bacterial and eukaryotic polysialic acid and can be used as probe for its detection. Glycoconjugate J. 18, 751-758 (2001). Clarify.

Response: It is true that the Aalto et al. paper deals the binding of the phage to polysialic acid but however to fixed cells and tissue sections. In the present study, we have mainly used live cells. Also as a difference our cells are from human origin. We have now indicated this by modifying the text (lines 76-78, 80-81, 105).

11. P4 L8: Polysialic acid is naturally found in which types of human cells?

Response: We have now included information on this in the beginning of the Results section (lines 78-79).

12. P4 L12: Perhaps some additional info could be provided on the cell line, especially for those that are not familiar with neuroblastoma cell line.

Response: According to the reviewer's suggestion, we have now added more information on the SK-N-SH cell line in the text (lines 86-87).

13. P4 L25: It should be indicated that this is bacterial polysialic acid. Is the phage receptor found in this prep? Could this explain the dissociation and the high-affinity reaction with the bacterial polysialic acid? The excess was determined how?

Response: We have now indicated the bacterial origin of soluble polysialic acid and revised the text to make the point more clear. We have also replaced the word 'excess' with 'a high concentration' (this concentration is known from previous experience, e.g. the Aalto et al. paper, to be high enough to inhibit phage-binding) (line 99).

14. P5 L1: Phage PK1A has the enzyme endosialidase and thus should still bind the polysialic acid (as indicated by Jakobsson et al. 2007). It seems that a one-hour incubation (at which temperature?) is enough for the phages to bind, hydrolyse the polysialic acid and be released. Perhaps testing another unrelated phage (as a negative control) without polysialic acid-binding capacity would be better?

Response: This is a good point. The phage with an active enzyme actually first binds but is then released due to polysialic acid hydrolyzing activity. Thus, as opposed to the phage containing catalytically inactive endosialidase it does not remain bound. We have now changed the text accordingly. The incubation

temperature has now also been added to the Methods section and legend of Fig. 1. (lines 101-102, 389, 696-697).

Testing an unrelated phage could theoretically be used as a negative control but there is no reason why such a phage should bind to polysialic acid. In contrast, a closely related phage that differs only two amino acid substitutions in the endosialidase is in our mind an ideal control to test the specificity of the interaction.

15. P5 L10/L22: After 6 hours, cell surface polysialic acid had disappeared? Why? This should be discussed.

Response: We have now discussed the reason for the disappearance of cell surface polysialic acid during the internalization process (lines 111-113).

16. P5 L14: Phages (plural). Check throughout the manuscript.

Response: We have corrected this and some other similar instances (e.g line 116).

17. P6 L3: Bacterial polysialic acid

Response: We have now added the missing word (line 130).

18. P6 L7: Phages are not viable per se. Consider revising. Check throughout the manuscript.

Response: We have now replaced the word 'viable' with 'intact' (line 172).

19. P6 L8: Any reason why the proteolytic treatments were not effective? Have you tried to increase the enzyme concentrations? This should be discussed.

Response: It is known that one primary function of the phage capsid is to protect the packaged genome from adverse environmental conditions such as proteases during its transfer between the host bacteria.

According to the reviewer's suggestion, we increased the concentration of subtilisin and proteinase K 10-fold (to 30 and 20 mg/ml, respectively) but detected only partial decrease in phage titers (19.6- and 1080-fold, respectively). We have now added information on this in the Results and Methods sections (lines 137-138, 446-447).

20. P6 L25: Why K1? According to P11 L20, the host of phage PK1A2 is strain EH954?

Response: We have now revised the text by removing the 'E. coli K1' (line 161).

21. P7 L3: 30%? Compared to the titer at 2h?

Response: We have now included this missing information in the text (line 165).

22. P7 L14: Considering that in the introduction you are mentioning DNA transfer between phage and human cells, I am surprised that the presence of phage DNA was not tested at 48 hours.

Response: We thank the reviewer for raising up this interesting matter. Similar questions related to phage DNA have also been raised by the Reviewer #2 and #3. To address this question, we have now added completely new results on the analysis of phage DNA which are depicted in Figure 6 and Supplementary Figures 1 and 2. The results have been discussed in the Results and Discussion sections (lines 181-215, 262-270).

3. P7 L21: Consider replacing head by capsid.

Response: As suggested by the reviewer, we have now replaced the word ‘head’ with ‘capsid’ (line 219).

24. P8 L5: Is it surprising to find that phages appeared to accumulate into large vesicles over time? This should be discussed.

Response: Our new experiments with endosomal markers, as suggested by Reviewer #2, indicate that PK1A2 phages are mostly delivered to lysosomes through the endosomal system during 24 h incubation (Fig. 4c,d and 6d,e). Thus, it is likely that the phage-loaded vesicles are matured to multivesicular bodies and then fused with lysosomes to become endolysosomes. In light of this, the accumulation is not unexpected. We have clarified the sentence related to the accumulation in the text (lines 230-232).

25. P10 L11: Is there such protein in phage PK1A2 (proteins covalently linked to the genome ends and contain eukaryotic nuclear localization signals that promote their accumulation in the nucleus)? If not, what is the likelihood of reaching the nucleus?

Response: There are no ORFs for terminal proteins in the genome sequence of PK1A2. However, as mentioned by Reviewer #3 (point 3), the presence of functional nuclear localization signals is not limited to terminal proteins. There is an example of a putative nuclear localization signal in one ORF of PK1A2 but any speculation on this is out of the scope of the present study. In an evolutionary timescale it is not known whether a specific nuclear localization signal is the requirement for a gene transfer event. We have now discussed this in the Discussion section (lines 263-265, 301-304).

26. P11 L24: Is there any polysialic acid left in the phage prep?

Response: Phages are purified by polyethylene glycol precipitation, CsCl density gradient ultracentrifugation and dialysis. Any polysialic acid should be removed by these treatments. In addition, the catalytic activity of

the PK1A phage would degrade any existing polysialic acid. For the PK1A2 phage, polysialic acid would prevent binding to the cells which is not the case.

27. P13 L9-12: Phage PK1A seems more efficient than the purified protein in hydrolyzing polysialic acid! This should be discussed.

Response: The endosialidase activities of the phage and the purified protein should not be compared because we did not determine the minimum amount needed for polysialic acid removal for either preparation. For the purified protein, we have used high enough concentration. The logic for choosing the concentration for the active phage was to use an equal number of particles as used for the inactive phage.

28. Fig 1: Phages were incubated for how long?

Response: We have now added the time (1 h) to the legend of Fig. 1 (line 696).

29. Fig 3e: Why not test the control PK1A phages containing catalytically active endosialidase at 4˚C?

Response: Thank you for the suggestion. This control has now added to the panel in Fig. 3e and has been discussed in the Results section (lines 130-131).

Reviewer #2 (Remarks to the Author):

This is a potentially interesting study that suggests that phage might be capable of mediating the horizontal transfer of DNA between prokaryotes and eukaryotes. Given the abundance of phage in our world and the fact that all metazoan systems appear to exist in close harmony with their own unique microbiome and associate phages, phage have ample opportunity to interact with eukaryotic cells. Yet there is very little if any reliable data indicating that they can transfer genetic material to eukaryotic cells. In this study the authors show that molecules very similar to bacterial phage receptors are expressed on mammalian cells and that these molecules can indeed bind phage particles and apparently cause them to be internalised into cells. It seems that the internalised phage enter intracellular vesicles but, over a period of 1 to 2 days, loose their integrity, as measured by their infectivity on cognate bacterial hosts. These are interesting observations that the authors suggest
establishes a case for phage being able to “cross the prokaryote–eukaryote barrier”, but in my view the study lacks key details that would enable the authors to make this claim with confidence.

The authors suggest that phage particles are taken up by receptor-mediated endocytosis, which may well be true. But if this is the case it would be relatively easy to identify the compartments in which internalised the particles are located by using well characterised markers for endocytic organelles and immunofluorescence analysis.

Response: Thank you for the suggestion. We have now performed immunofluorescence analysis with three widely-used endosomal markers, EEA1, LAMP1 and Lysotracker. The data is shown in Figs 4 and 6 of the revised manuscript and discussed in the main text (lines 149-154, 209-215, 261-270).

Although the authors show phage uptake, crucially they do not show transfer of phage DNA to cells. Although phage appear to be taken up into vesicles, they are still separated from the cytoplasm by a vesicle membrane, so the notion that they reach the 'cell interior' (line 184) is not really established. Moreover, as the internalised phage gradually loose integrity, is it not just as likely they are degraded in lysosomes? For this study to really make an impact it will be crucial to show that phage DNA is delivered into the cell cytoplasm or nucleus.

Response: We thank the reviewer for this thoughtful criticism and for highlighting these important issues. We definitely agree the need for additional experimental support to strengthen our conclusion. Regarding the concerns, the other reviewers also suggested the examination of the fate of phage DNA within cells. As mentioned above (Reviewer #1 point 22), we have now added new results on this (Fig. 6 and Supplementary Fig. 1 and 2).

The point of the paper is to show that the phage is able to pass the cell membrane barrier and become localized inside the cell. We agree that the 'cell interior' is a vague term and have therefore changed the text (lines 240, 301).

The lysosomal localization has been addressed by the marker studies (see above).

The question of reaching the cytoplasm or nucleus have been addressed in the response to Reviewer #1 (point 25) and in the Discussion section (lines 261-270, 301-304).

Minor points:-

Line 83: Epifluorescence imaging lacks the resolution necessary for the authors to claim that receptors and phage are 'co-localised'. They should say 'have similar distributions'.

Response: We have now revised the sentence as suggested (line 91).

Line 93: Why do the authors say 'apparently due to their polysialic acid cleaving activity', has this been established?

Response: This was admittedly not entirely clear and we have therefore modified the text and added a reference (lines 101-102).

Line 99: From this data the authors can't say whether the phage are in the cytoplasm or within vesicles. All that they can say is that they appear to have been internalised.

Response: This is indeed true and we have therefore removed the word ‘cytoplasm’ and replaced it with ‘cell’ (line 107).

Fig 6a: The right hand 24 hr panel should be removed. It’s impossible to say whether the vacuole contains phage particles or is a multivesicular body.

Response: As advised, we have now removed this part in Fig. 6a.

Reviewer #3 (Remarks to the Author):

The article by Jukka Finne and collaborators provide solid evidences of the entrance of PK1A2 phage into polysialic acid-containing human cells. The experiments are clear and I do believe that all negative and positive controls demonstrate the specific binding and internalization of the phage intro the mammalian cells. This novel piece of data further supports previous hypotheses about the role of bacteriophages as vectors for prokaryotes-to-eukaryotes horizontal gene transfer, a phenomenon that have been somewhat controversial. Therefore, I am sure that the paper will raise the interest of researchers from both prokaryotic and eukaryotic side as well as evolutionary and computational biologist.
I have some comments that hopefully may help to strength the manuscript and clarify some aspects of the Discussion regarding the biological importance of these findings.

Response: We would like to thank the reviewer for the encouraging comments.

1. The authors showed that phage infectivity drops after 48 h inside the eukaryotic endocytic pathway, which would be consequence of the destabilization of the viral particles. It would be very interesting to examine this more deeply. In particular, I wonder whether this virions deactivation eventually leads to viral DNA partial leakage or release, which would be required to continue its journey to the cell nucleus.

Response: We thank the reviewer for bringing up this suggestion to strengthen our conclusion. We have now performed these experiments. The results are discussed above in our response to Reviewer #1 (point 22).

2. Regarding the discussion of the biological relevance of bacterial viruses inside eukaryotic cells, I agree that HGT is a very likely hypothesis and it is well presented in the discussion. However, once phage DNA eventually reach the cell nucleus, either in a somatic or non-somatic cell, other effects with biological meaning may occur and should be considered in the Discussion section. For instance, modulation of immune system by phage genes has been already suggested (ref. 45). The presence of prokaryotic sequences in tumor cells have been also showed (Riley D.R. et al. Plos Comput Biol; 9(6) 2013).

Response: We thank the reviewer for raising up these issues and giving the references. These points have now been discussed in the Discussion section (lines 266-270, 292).

3. Finally, as the authors mentioned, the nuclear envelope constitutes an additional barrier that only very small DNA molecules can overcome. Phage terminal proteins have been shown to enhance gene delivery by means of functional NLSs. However, although widespread among different viral families and other diverse replicons, protein-primed DNA replication is not a very frequent mechanism. Interestingly, other phage proteins have been shown to contain functional NLSs, like P1 Cre recombinase and other DNA binding proteins, that may also favor nuclear internalization of phage DNA. I wonder if there may be putative NLSs-containing proteins among the PK1A or PK1A2 proteome.

Response: The possible presence of functional nuclear localization signals in PK1A2 and PK1A genome sequence is an interesting question. As mentioned above (Reviewer #1 points 7 and 25), we sequenced the genomes of PK1A2 and PK1A and searched for terminals proteins and nuclear localization signals. Please see our responses above.

Other minor points:

1. Lane 59. This statement seems to me somewhat misleading. As the authors mentioned in the immediately above sentence (lane 57), phage genes are functional in eukaryotic cells, which is a strong “experimental evidence” that phages (at least in those cases) indeed entered the eukaryotic cell. I believe that the authors should highlight the lack of a direct evidence that show the detailed mechanism of virion binding and subsequent internalization in a nonphagocytic cell, as has been demonstrated in this work.

Response: We thank the reviewer for the thoughtful suggestion. We have now rephrased the corresponding sentence and also made other changes according to the advice giving (lines 58-60, 250-252).

2. Lane 236. “Infected cells” is not formally correct, since the phage are not replicating within the eukaryotic cells. One might say abortive infection, but I believe that it would be better to avoid this term.

Response: We agree with the reviewer's comment. We have now removed the word ‘infected’ from the revised manuscript (line 301).

Reviewers' comments:

Reviewer #1 (Remarks to the Author):

The revised manuscript has addressed a significant number of concerns, and is certainly stronger for it. The well-thought out rebuttal is also appreciated. Still, there are a few additional comments that need to be

1. L25: Suggestion: "… which share some structural similarities with a specific phage receptor.
2. Line 26: Replace "DNA portion" by "genome".
3. Line 27: "Phages were…"
4. Line 28: Replace "survived" by "persist".
5. Line 28: "…human cell viability".
6. Line 34:"…organisms for the completion…".
7. Line 37: Replace "encapsulate" by "package".
8. Line 73: " The bacterial receptor…".
9. Line 111: "…binding induces… leads to progressive disappearance of polysialic…". If there are no phages, do you see the disappearance of polysialic acid complex over time? Perhaps this control was done already and if so, should be added.
10. L136: Remove "at all".
11. L156: Replace "Survival" per "Persistence". Change title of Figure 5 too.
12. L157: Replace "intact" per "infectious".
13. L167: "inside the kSK-N-SH cell, …".
14. L172: Replace "intact" per "infectious".
15. L176: More info should be provided here on the cell viability assays as it seems different than the previous assays in Figure 5. How were the various titers of phages added? Are these titers of internalized phages? If so, how were you able to obtain such high titer phage internalized? If not, perhaps these should be presented only with internalized phage titers. Otherwise, I am not sure what is the exact meaning of the assay. Pretty obvious that phage won't kill cells from the outside. Please clarify.
16. L203: Considering the claim below, this must be shown.
17. L206-207: This does NOT show that it is incorporated into the nuclear DNA. At best, it may show that it reaches the nucleus. Or I am missing something?
18. L238: Have these been really observed?
19. L265-266: I don't think this was shown here.
20. L266-268: Not sure this is needed. We are continuously exposed to foreign DNA. Not sure what is the link with an immune response and internalization of phages. I think the lines 268-270 are more appropriate.
21. L273:"…acid at the surface of the eukaryotic cell lines used in this study explains…".
22. L274: Is the bacterial host also internalized as well? If so, a very nice experiment would be to see if a phage-infected bacterial cell could be used as a cargo to deliver phages inside the eukaryotic cell. For another study.
23. L276: One transition sentence that could be added here is that the observations made in your study was done with one specific phage and one particular human neuroblastoma cell line in vitro. The take home message should not be that all the phages can do this and all cells can take up phages.
24. L301: Is transport the accurate term here?
25. L301: Cell membrane? is it nucleus? Somewhat confused by the sentence.
26. L272-304: This paragraph seems redundant with what was already discussed.
27. L322: I am not an expert in human cell line but would the presence of high glucose lead to more polysialic acid production?
28. L330: To be done before acceptation.
29. L389: I understand that the cells were fixed but I wonder why the cells were not incubated at 37˚C?
30. L415: At which temperature? In fact, temperatures are missing in many places in the M+M

section. Check throughout the section.
31. L460: phages
32. L461: cells
33. L486: Indicate the phage titers tested.
34. Fig. 1b. While it is indicated in the legend, I would still add below “Control” that these are kSK-N-SH cells.
35. Fig. 8. Are these thin sections as well?

Reviewer #2 (Remarks to the Author):

Lehti et al. have resubmitted their paper describing a possible mechanism for phage-mediated prokaryote to eukaryote gene transfer. The authors have addressed most of my comments and show a fairly compelling case for an endocytic mechanism being involved, though they have not identified which specific endocytic pathway(s) is/are involved. In many respects, this uptake is reminiscent of the mechanisms that many eukaryotic viruses use to gain access to cells. The main thing that concerns me is that although the authors show some evidence for phage-derived nucleoside analogues being incorporated into the cellular nuclear DNA, there is no evidence for phage- associated sequences being transferred. Given this is the major point of the paper, they have not actually shown that endocytosis of phage can result in cells acquiring new genetic sequences, regardless of how likely this is to be the case.

Minor comments: -

Title and line 25: Change ‘to’ to ‘into’.

Line 27: Change ‘the’ to ‘its’.

Line 28: Delete ‘to’.

Line 29: Change ‘The interaction discovered may represent’ to ‘Our results suggest’.

Line 30: Change ‘to the completion of’ to ‘to complete their’.

Lines 60-62: The phrasing should be improved for clarity. ‘Here, we show interaction of bacteriophage with a cell surface glycan, followed by internalization of the phage across the eukaryotic cell membrane inside a mammalian cell’. The authors show internalization of phage into endocytic vesicles, but they do not should ‘internalization across the eukaryotic cell membrane inside a mammalian cell’. It’s not even clear what this means; translocation across the plasma membrane (which is not inside the cell) or translocation across an endosomal membrane? If they mean the later, they do not show it. The word ‘conceivably’ in the following sentence perhaps underplays the latter scenario, as this penetration from endocytic organelles is a pathway well established for eukaryotic cell viruses.

Line 71: Change ‘has been’ to ‘was’.

Line 91: Change ‘and had similar distribution with’ to ‘and had a similar distribution to’.

Line 92: Change ‘SH-SY5Y cells also’ to ‘SH-SY5Y cells that also express’

Line 93: Change ‘BJ fibroblasts containing little or no polysialic acid’ to ‘BJ fibroblast, which contain little or no polysialic acid,’.

Line 97: Change ‘an enzyme specifically degrading polysialic acid’ to ‘an enzyme that specifically

degrades polysialic acid'.

Fig. 2: Can the authors give an idea of the number of particles per cell? Currently they just give total pfu added to cells. What is the particle to pfu ratio?

Fig. 4a: The acid wash panels seem unnecessary: Acid wash doesn't work, it is stated clearly enough in the text.

Line 140/141: Delete second 'cell surface" it redundant

Line 213/214: The authors suggest that some of the internalized phage is not in late endosomal/lysosomal compartments after 24hrs at 37°C. They should be cautious with this conclusion as it may just be that the markers used have failed to access some of the lysosomal organelles.

Line 237: Delete 'of'.

Line 263: Change 'an outmost rare event' to a 'rare event'

Line 266: To avoid an misunderstanding through using 'recycling', the authors should say 'incorporation into cellular DNA'.

Line 268: Do they see any activation of TLRs or innate immune pathways?

Line 273: Change 'both cells' to 'both cell types'.

Line 284: Change 'also be generated by incidence' to 'may be generated naturally'

Line 303: Delete 'of'.

Reviewer #3 (Remarks to the Author):

The authors have answered all the questions raised and also provide new results that complete a round history. The new version of the manuscript is sound and the possible role of phages as vehicles for HGT is presented as a compelling and plausible hypothesis.
My only concern is that the new experiment in Figure 6 is not conclusive regarding the fate of the viral DNA within the eukaryotic cell and it is only briefly discussed. Quite rightly, the authors point out that rare events on the evolutionary scale are likely to be very difficult to detect under standard experimental conditions. However, a more direct technique, such as expression of a viral marker or detection of viral DNA sequences by FISH within the nucleus, could also have been indicated.

Responses to reviewers

We thank the Editor and the Reviewers once more for giving their valuable comments on the manuscript. We have performed all the suggested additional experiments, most notably the FISH detection of internalized phage DNA. The constructive criticism helped us to still improve the manuscript. We hope that the manuscript is now ready for publication.

The changes made are listed below and highlighted in the revised manuscript.

Reviewer #1 (Remarks to the Author):

The revised manuscript has addressed a significant number of concerns, and is certainly stronger for it. The well-thought out rebuttal is also appreciated. Still, there are a few additional comments that need to be

> Response: We thank the Reviewer for the kind comment and all the detailed suggestions to improve the manuscript.

1. L25: Suggestion: "… which share some structural similarities with a specific phage receptor.

> Response: We have revised the text as outlined by the Reviewer (lines 25-26).

2. Line 26: Replace "DNA portion" by "genome".

> Response: We have done this and also slightly simplified the sentence (lines 26-27).

3. Line 27: "Phages were…"
4. Line 28: Replace "survived" by "persist".

> Response: We appreciate these suggestions and have done the changes (lines 27, 28).

5. Line 28: "…human cell viability".

> Response: We have now indicated in the sentence that the cell is of human origin (lines 27-29).

6. Line 34:"…organisms for the completion…".

> Response: We have changed this taking also in consideration the suggestion of Reviewer #2 (line 34).

7. Line 37: Replace "encapsulate" by "package".
8. Line 73: " The bacterial receptor…".

Response: We have now done the suggested changes (lines 37, 72).

9. Line 111: "…binding induces… leads to progressive disappearance of polysialic…". If there are no phages, do you see the disappearance of polysialic acid complex over time? Perhaps this control was done already and if so, should be added.

Response: We have now performed this experiment and included the results as Supplementary Fig. 1 and mentioned the results in the text (lines 109-110).

10. L136: Remove "at all".
11. L156: Replace "Survival" per "Persistence". Change title of Figure 5 too.
12. L157: Replace "intact" per "infectious".
13. L167: "inside the kSK-N-SH cell, …".
14. L172: Replace "intact" per "infectious".

Response: We appreciate these suggestions and have done the changes (lines 136, 156, 157, 167, 172, 782).

15. L176: More info should be provided here on the cell viability assays as it seems different than the previous assays in Figure 5. How were the various titers of phages added? Are these titers of internalized phages? If so, how were you able to obtain such high titer phage internalized? If not, perhaps these should be presented only with internalized phage titers. Otherwise, I am not sure what is the exact meaning of the assay. Pretty obvious that phage won't kill cells from the outside. Please clarify.

Response: Admittedly the description was not entirely clear and we have now clarified it in the main text as well as in the figure legend (lines 176-179, 794).

16. L203: Considering the claim below, this must be shown.

Response: We have now added the results of the EdU and BrdU stainings as compared to DAPI (Supplementary Fig. 4 and 5).

17. L206-207: This does NOT show that it is incorporated into the nuclear DNA. At best, it may show that it reaches the nucleus. Or I am missing something?

Response: We appreciate this point and have now modified the text accordingly (lines 217-221, 274-275). We also made additional controls to verify that the nuclear localization of phage-derived nucleosides depends on the initial polysialic acid-mediated internalization of the phages (Supplementary Fig. 4, third columns).

18. L238: Have these been really observed?

Response: We have now changed the sentence accordingly (lines 243-244).

19. L265-266: I don't think this was shown here.

Response: We have now addressed this as described above, point 17.

20. L266-268: Not sure this is needed. We are continuously exposed to foreign DNA. Not sure what is the link with an immune response and internalization of phages. I think the lines 268-270 are more appropriate.

Response: We have change the text to indicate that we discuss foreign intracellular DNA. We have also added a citation to a recent review (Ref. 49) discussing the immunomodulatory effects of phages (lines 275-277).

21. L273:"...acid at the surface of the eukaryotic cell lines used in this study explains...".

Response: We have now changed the sentence as instructed (line 281).

22. L274: Is the bacterial host also internalized as well? If so, a very nice experiment would be to see if a phage-infected bacterial cell could be used as a cargo to deliver phages inside the eukaryotic cell. For another study.

Response: This is a very interesting point indeed and could be a topic of another study.

23. L276: One transition sentence that could be added here is that the observations made in your study was done with one specific phage and one particular human neuroblastoma cell line in vitro. The take home message should not be that all the phages can do this and all cells can take up phages.

Response: Thank you very much for this comment. We have changed the text taking this in consideration (lines 285-286).

24. L301: Is transport the accurate term here?
25. L301: Cell membrane? is it nucleus? Somewhat confused by the sentence.
26. L272-304: This paragraph seems redundant with what was already discussed.

Response: We have now changed 'transport' to 'penetration' and clarified the sentences. The redundancy has been now removed (lines 311-313). We thank the Reviewer for the suggestions.

27. L322: I am not an expert in human cell line but would the presence of high glucose lead to more polysialic acid production?

Response: It is correct that glucose level may influence cell surface glycosylation. DMEM was originally formulated with low glucose (1 g/l) but nowadays DMEM typically contains higher levels of glucose. The "high glucose" condition reflects typical cell culture media. To be more exact, we have now indicated the concentration of glucose (4.5 g/l) in the DMEM used (line 331).

28. L330: To be done before acceptation.

Response: We have now submitted the PK1A2 genome sequence to GenBank, accession number MG004687 (lines 339-340, 553). It will be released after the publication of the present study.

29. L389: I understand that the cells were fixed but I wonder why the cells were not incubated at 37˚C?

Response: When we are dealing with live cells, it is relevant to perform the experiments at 37 °C. However, with fixed cells, it is standard procedure to do the stainings for practical reasons at room temperature. For clarity, we have now added the word 'fixed' to the appropriate position (line 399).

30. L415: At which temperature? In fact, temperatures are missing in many places in the M+M section. Check throughout the section.

Response: We agree absolutely. The missing temperatures have now been added (lines 345, 348, 354, 359, 364, 370, 376, 397, 398, 409, 412, 421, 426, 427, 429, 437, 439, 447, 448, 450, 512, 529, 532, 536).

31. L460: phages
32. L461: cells
33. L486: Indicate the phage titers tested.
34. Fig. 1b. While it is indicated in the legend, I would still add below "Control" that these are kSK-N-SH cells.
35. Fig. 8. Are these thin sections as well?

Response: All these changes have been made according to the advices given (lines 491, 492, 517, 826-827 and Fig. 1b).

Reviewer #2 (Remarks to the Author):

Lehti et al. have resubmitted their paper describing a possible mechanism for phage-mediated prokaryote to eukaryote gene transfer. The authors have addressed most of my comments and show a fairly compelling case for an endocytic mechanism being involved, though they have not identified which specific endocytic pathway(s) is/are involved. In many respects, this uptake is reminiscent of the mechanisms that many eukaryotic viruses use to gain access to cells. The main thing that concerns me is that although the authors show some evidence for phage-derived nucleoside analogues being incorporated into the cellular nuclear DNA, there is no evidence for phage- associated sequences being transferred. Given this is the major point of the paper, they have not actually shown that endocytosis of phage can result in cells acquiring new genetic sequences, regardless of how likely this is to be the case.

Response: We would like to express our appreciation for the Reviewer's careful reading of the manuscript. The Reviewer is correct in that the transfer of phage-associated sequences is not shown. The main point of the paper is that bacteriophages can pass the eukaryotic cell membrane barrier and be internalized into the cell using a specific receptor. This finding may be relevant in the tracing of the origin of prokaryotic sequences in eukaryotic genomes, which already for some time has intrigued investigators in the field. This has been taken up as a matter of discussion in our manuscript. It should be also noted that the discussed horizontal gene transfer is expected to be a rare event during evolution, highly unlikely to be observed under conventional laboratory conditions. We would like to point out that there are also other biological consequences of the internalization such as the reduced cell surface expression of polysialic acid and possible cell responses to foreign DNA.

On the whole, we feel that our observation of phage penetration and internalization into the eukaryotic cells is surprising and of interest in many different fields.

We have now added a sentence to Discussion to pointing out the change affecting cell surface polysialic acid expression (lines 266-268).

Minor comments: -

Title and line 25: Change 'to' to 'into'.
Line 27: Change 'the' to 'its'.
Line 28: Delete 'to'.
Line 29: Change 'The interaction discovered may represent' to 'Our results suggest'.
Line 30: Change 'to the completion of' to 'to complete their'.

Response: All these changes have been made according to the advices given (lines 5, 25, 27, 28, 29, 34).

Lines 60-62: The phrasing should be improved for clarity. 'Here, we show interaction of bacteriophage with a cell surface glycan, followed by internalization of the phage across the eukaryotic cell membrane inside a mammalian cell'. The authors show internalization of phage into endocytic vesicles, but they do not should 'internalization across the eukaryotic cell membrane inside a mammalian cell'. It's not even clear what this means; translocation across the plasma membrane (which is not inside the cell) or translocation across an endosomal membrane? If they mean the later, they do not show it. The word 'conceivably' in the following sentence perhaps underplays the latter scenario, as this penetration from endocytic organelles is a pathway well established for eukaryotic cell viruses.

Response: This sentence was admittedly not clear and we have now rewritten it as outlined by the Reviewer (lines 61-62).

Line 71: Change 'has been' to 'was'.
Line 91: Change 'and had similar distribution with' to 'and had a similar distribution to'.
Line 92: Change 'SH-SY5Y cells also' to 'SH-SY5Y cells that also express'

Line 93: Change ‘BJ fibroblasts containing little or no polysialic acid’ to ‘BJ fibroblast, which contain little or no polysialic acid,’.
Line 97: Change ‘an enzyme specifically degrading polysialic acid’ to ‘an enzyme that specifically degrades polysialic acid’.

Response: We have now done the suggested changes (lines 70, 91, 92, 93, 97).

Fig. 2: Can the authors give an idea of the number of particles per cell? Currently they just give total pfu added to cells. What is the particle to pfu ratio?

Response: In Fig. 6c, we have counted the number of phage vesicles per cell (about 17 vesicles at 24 h). Considering that each vesicle may contain several phages (Fig. 8), there are tens of phage particles in each cell. In general, the particle to pfu ratio is considered to be close to 1. We have used phages purified by CsCl density gradient ultracentrifugation, which removes empty capsid particles lacking DNA.

We have added the information that each vesicle may contain several phage particles to the legend of Fig. 6c (lines 804-805).

Fig. 4a: The acid wash panels seem unnecessary: Acid wash doesn’t work, it is stated clearly enough in the text.

Response: We have now removed the acid wash results from Fig. 4. They are now provided as Supplementary Fig. 2.

Line 140/141: Delete second ‘cell surface” it redundant

Response: We have now deleted the second ‘cell surface’ (line 140).

Line 213/214: The authors suggest that some of the internalized phage is not in late endosomal/lysosomal compartments after 24hrs at 37°C. They should be cautious with this conclusion as it may just be that the markers used have failed to access some of the lysosomal organelles.

Response: We have now pointed out this reservation (lines 208-209).

Line 237: Delete ‘of’.
Line 263: Change ‘an outmost rare event’ to a ‘rare event’

Response: We have now done these changes (Lines 243, 273).

Line 266: To avoid an misunderstanding through using ‘recycling’, the authors should say ‘incorporation into cellular DNA’.

Response: We have now changed this but avoided the use of ‘incorporation’ as instructed by Reviewer #1 point 17. (lines 274-275).

Line 268: Do they see any activation of TLRs or innate immune pathways?

Response: This would be indeed be an interesting line of investigation but is clearly a matter of another study.

Line 273: Change ‘both cells’ to ‘both cell types’.
Line 284: Change ‘also be generated by incidence’ to ‘may be generated naturally’
Line 303: Delete ‘of’.

Response: We have now done these changes, for the last one we removed the whole sentence (Reviewer #1, point 26.) (lines 282, 293).

Reviewer #3 (Remarks to the Author):

The authors have answered all the questions raised and also provide new results that complete a round history. The new version of the manuscript is sound and the possible role of phages as vehicles for HGT is presented as a compelling and plausible hypothesis.
My only concern is that the new experiment in Figure 6 is not conclusive regarding the fate of the viral DNA within the eukaryotic cell and it is only briefly discussed. Quite rightly, the authors point out that rare events on the evolutionary scale are likely to be very difficult to detect under standard experimental conditions. However, a more direct technique, such as expression of a viral marker or detection of viral DNA sequences by FISH within the nucleus, could also have been indicated.

Response: Thank you very much for the suggestion, we have now done the FISH experiments (Fig. 6f, lines 212-215).

REVIEWERS' COMMENTS:

Reviewer #3 (Remarks to the Author):

I have no further concerns regarding the clarity, novelty or interest of the results.

Response to issues raised by the referees

REVIEWERS' COMMENTS:

Reviewer #3 (Remarks to the Author):

I have no further concerns regarding the clarity, novelty or interest of the results.

Response: We appreciate very much this positive statement and thank the reviewer for his/her work to improve the manuscript.
